# Screening of β1- and β2-Adrenergic Receptor Modulators through Advanced Pharmacoinformatics and Machine Learning Approaches

**DOI:** 10.3390/ijms222011191

**Published:** 2021-10-17

**Authors:** Md Ataul Islam, V. P. Subramanyam Rallabandi, Sameer Mohammed, Sridhar Srinivasan, Sathishkumar Natarajan, Dawood Babu Dudekula, Junhyung Park

**Affiliations:** 1 3BIGS Omicscore Pvt. Ltd., 1, O Shaughnessy Rd, Langford Gardens, Bengaluru, Karnataka 560025, India; ataul@3bigs.com (M.A.I.); subramanyam@3bigs.com (V.P.S.R.); sameer@3bigs.com (S.M.); sridhar@3bigs.com (S.S.); dawood@3bigs.com (D.B.D.); 2 3BIGS Co., Ltd., 156, Gwanggyo-ro, Yeongtong-gu, Suwon-si 16506, Korea; sathish@3bigs.com

**Keywords:** cardiovascular diseases, β-adrenergic receptors, virtual screening, machine learning, similarity search, MD simulation

## Abstract

Cardiovascular diseases (CDs) are a major concern in the human race and one of the leading causes of death worldwide. β-Adrenergic receptors (β1-AR and β2-AR) play a crucial role in the overall regulation of cardiac function. In the present study, structure-based virtual screening, machine learning (ML), and a ligand-based similarity search were conducted for the PubChem database against both β1- and β2-AR. Initially, all docked molecules were screened using the threshold binding energy value. Molecules with a better binding affinity were further used for segregation as active and inactive through ML. The pharmacokinetic assessment was carried out on molecules retained in the above step. Further, similarity searching of the ChEMBL and DrugBank databases was performed. From detailed analysis of the above data, four compounds for each of β1- and β2-AR were found to be promising in nature. A number of critical ligand-binding amino acids formed potential hydrogen bonds and hydrophobic interactions. Finally, a molecular dynamics (MD) simulation study of each molecule bound with the respective target was performed. A number of parameters obtained from the MD simulation trajectories were calculated and substantiated the stability between the protein-ligand complex. Hence, it can be postulated that the final molecules might be crucial for CDs subjected to experimental validation.

## 1. Introduction

G-protein-coupled receptors (GPCRs) are a group or superfamily of membrane proteins that respond to a variety of extracellular signals and thereby regulate a number of physiological and pathological processes [1,2,3,4,5,6,7]. According to the composition of amino acid sequences, the human GPCR family is categorized into four subfamilies, namely: A (rhodopsin), B (secretin and adhesion), C (glutamate), and F (Frizzled) [1]. It is quite interesting to mention that about one-third of all clinically used medicines’ targets are GPCRs [6]. Therefore, GPCR target palettes have sparked a renaissance in GPCR pharmacology by allowing researchers to screen the GPCRs of specific therapeutic interest for the subsequent drug development process. GPCRs actively participate in various signal transduction pathways across cell membranes in response to extracellular stimuli like small molecular contacts, protein, peptide, hormones, ions, and exposure to light. There are several selective GPCRs that are abundantly expressed in cardiovascular tissues to maintain cardiovascular homeostasis, including adrenergic, adenosine, endothelin, and angiotensin receptors. Importantly, adrenergic receptors are accountable for decoding chemical information from the sympathetic nervous system into cardiovascular responses [8]. Notably, GPCR-mediated adrenergic dysregulation has been linked to the onset and progression of serious cardiovascular disorders, eventually leading to coronary artery failure [9]. Therefore, GPCRs have emerged as one of the most important US Food and Drug Administration (FDA)-approved drug targets for managing cardiovascular pharmacotherapy [9]. Tremendous efforts have been made in the past decades to better understand GPCR kinase’s role in the pathophysiology of cardiac diseases. Earlier, a lack of actual active structural conformation that induces G-protein-mediated signaling might have slightly hampered the actual understanding of the underlying activation mechanism for GPCRs proteins. However, advancements in high-throughput protein engineering and analytical techniques have solved a number of conformational states for many GPCRs proteins, including adrenergic receptors [10]. The available crystal structure of GPCRs proposes new opportunities for structure-based drug design (SBDD). In particular, to increase the formation of thermostabilize crystal connections, the enzyme T4 lysozyme (T4L) was inserted into intracellular loop 3 of the receptor [11]. From this perspective, we considered the two important beta-adrenergic receptors (β-AR) for the SBDD study. The β1-AR of the GPCR kinases family, which are highly associated with the overall regulation of cardiac function [12], are three human β-ARs, namely β1_,_ β2, and β3-AR, which share 51% sequence identity [13]. Moreover, it has been previously reported that genetic polymorphisms in all three subtypes of β-ARs (β1, β2, and β3 AR) are also associated with several non-communicable diseases, including cardiovascular disorders, chronic obstructive pulmonary disease, asthma, and obesity [14,15,16]. Precisely, the activation or stimulation of adrenergic receptors are the primary key regulators of heart rate and myocardial contractility [12]. It has been said that β-blockers *viz.* antagonists and inverse agonists (drugs that impede β1-AR and β2-AR signaling) are used to modify heart function. A number of research studies highlighted the identification of either agonist, partial agonists, or weak partial agonists of β-AR [11,17,18,19,20,21,22]. However, such investigation is facing a lot of challenges regarding the identification and development of selective β-blockers mainly because of allosteric factors and conformational flexibility [23]. Therefore, the discovery of subtype-selective drugs for β-AR remains a major hurdle in pharmaceutical industries, making them attractive drug targets for managing many chronic cardiovascular diseases.

The structural organization demonstrated that among the total of 313 amino acid residues of β1-AR, 15 amino acid side chain residues form the four transmembrane-helices and two extracellular loops at the ligand-binding pocket of β1-AR [13]. Precisely, the presence of two disulfide bonds helps in stabilizing the loop conformation. It has been confirmed that a similar type of interaction was observed for both β1- and β2-AR when interacted with β-blockers like cyanopindolol (antagonist) and carazolol (inverse agonist) [13]. Although human β1- and β2-AR possesses 67% identity within their transmembrane regions, their amino acid residues that directly surround the active site or ligand-binding pocket are identical [13]. The different distinct conformations of the active and inactive state of β1–AR bound to cyanopindolol have also been recently demonstrated [24]. The overall extracellular loop (ECL-3) structure of β1-AR has shown high sequence conservation with β2-AR (Cα rmsd 0.8 Å). Moreover, the cytoplasmic loop structures (CL2 and CL3) are thought to be crucial in the binding, selectivity, and activation of G proteins in all GPCRs including β1- and β2-AR. In β1-AR, the CL2 forms a tiny α-helix with amino acid residues extending from Pro146 to Leu152, whereas in the β2-AR structure, this loop forms as an extended conformation. Most importantly, the selectivity of the ligand-binding site is represented by extracellular loop 2 (ECL2) consisting of 15 amino acid residues at the ligand-binding pocket [13].

The β2-AR is mostly found in smooth muscle in the body. A study has demonstrated the overall β2-AR structural topology that also consists of seven transmembrane helices forming a helical bundle: helix I consisting of residues 29 to 60, helix II extending from residues 67 to 96, helix III 103 to 136, helix IV 147 to 171, helix V 197 to 229, helix VI 267 to 298, and helix VII 305 to 328. Except above there are other intermediate residues, which form loops consisting of intracellular loops (ICLs) and extracellular loops (ECLs) of β2-AR: the ICL1 extending from amino acids 61 to 66, ECL1 residues 97 to 102, ICL2 residues 137 to 146, ECL2 residues 172 to 196, ICL3 residues 230 to 266, and ECL3 residues 299 to 304. Each of the transmembrane segments’ helices II, V, VI, and VII has a proline-induced kink at the conserved locations. These kinks are required for structural rearrangements and thereby helps in G protein effectors’ activation [25].

The current age of drug development includes not only a straightforward rational approach, but also the use of advanced computational techniques to expedite the overall developmental process, reduce cost and time, and ensure the safety effectiveness of identified compounds. The chemical space available to the scientific community necessitates comprehensive compound screens for identifying a potential molecule that might strongly bind to a target of interest. Considering such an attractive and commendable way for drug discovery research, the present comprehensive study focused on identifying potential small chemical entities through computational screening of ~99 million chemical compounds, which can possibly and strongly interact with both β1- or β2-AR GPCR. The PubChem database [26] is known as the world’s largest publicly available chemical repository. Herein, the PubChem database was considered to screen out the potent compounds employing a set of advanced computational techniques. Particularly, combining several ever-advancing tools and technologies like drug-likeness-based filtration, molecular docking and dynamics simulation, and machine learning (ML) analysis for descriptor selection and validation, substructure search against DrugBank and ChEMBL, and binding free energy estimation for identified compounds was employed as the key steps in the present study. Based on the extensive screening protocol followed by the identification of potential small molecules, modulators of β1- and β2-AR have been found to be pharmacologically most potent compounds. Hence, the main objective of the current study was to explore the potential chemical entities through computational drug discovery approaches for therapeutic applications in cardiovascular diseases.

## 2. Results and Discussion

Molecular docking-based virtual screening was performed to identify the potential inhibitors/modulators of β1- and β2-AR for therapeutic application in cardiovascular diseases. Prior to molecular docking for screening of chemical databases, it is necessary to validate the protocol. The self-docking approach was used for both the β1- and β2-AR, in which the co-crystalized ligand was re-drawn and docked at the same coordinates where it was originally bound. The best pose of β1- and β2-AR was superimposed to the respective original co-crystal ligand. The RMSD of the superimposed crystal structure was found to be 1.874 and 1.960 Å for β1- and β2-AR, respectively. Moreover, the binding interaction and orientation of self-docked ligands were also found to be comparable to the co-crystal ligands. Superimposed root-mean square deviation (RMSD) value and binding interaction analyses of both β1- and β2-AR clearly validated and indicated that the considered docking protocol might be useful to generate an orientation of molecule in molecular docking similar to the experimental conformation. Superimposed structures of the self-docked and co-crystal ligand for both β1- and β2-AR are given in Figure S1 (Supplementary Materials data).

### 2.1. Virtual Screening

**Figure 1:**
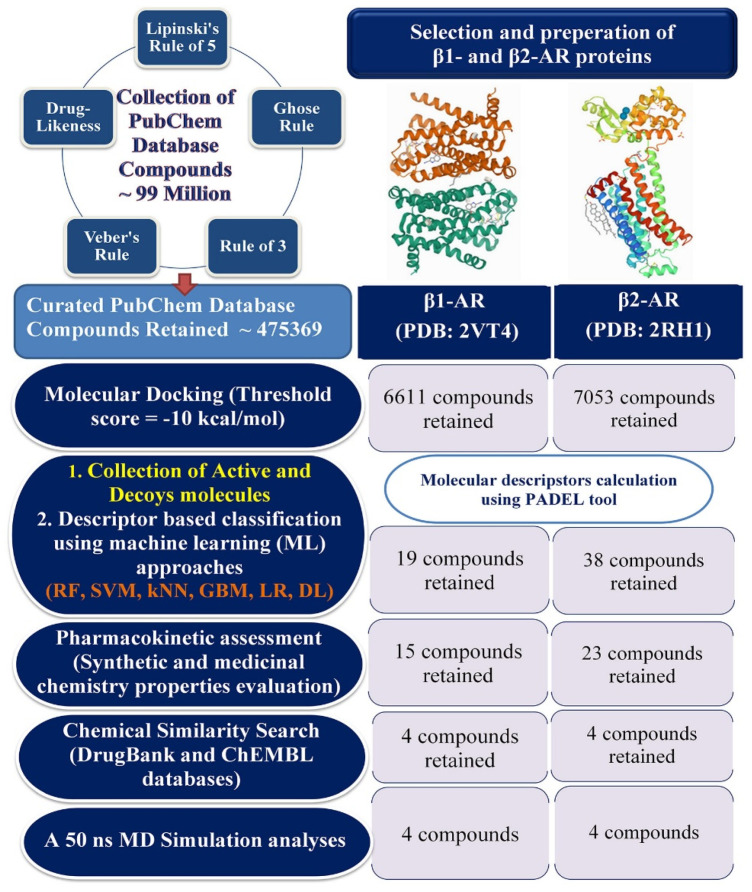
Workflow of virtual screening of the PubChem database against β1- and β2-AR. RF: Random Forest; SVM: Support Vector Machine; kNN: k-Nearest Neighbors; GBM: Gradient Boosting Machine; LR: Logistic Regression; DL: Deep learning.

The entire PubChem database was screened through a number of criteria included as the satisfaction of Lipinski’s rule of five (LoF), Rule of three, Ghose rule, Veber’s rule, and drug likeness properties. After successful filtration through the above-mentioned rules, a total of 475,369 molecules were retained. Above entire set of screened molecules were docked into the active site of both β1- and β2-AR simultaneously. The stepwise workflow employed for the screening purposes on the collected PubChem database molecules is given in Figure 1.

**Figure 2:**
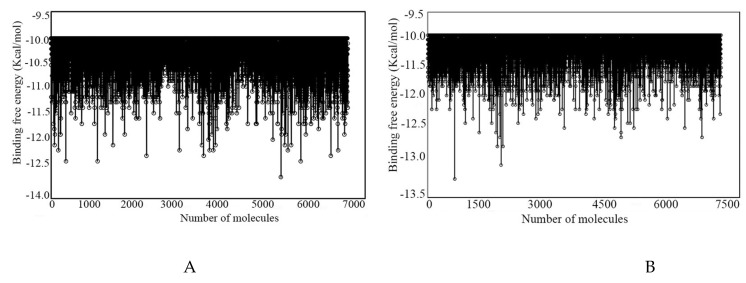
Binding energy of (**A**) β1- and (**B**) β2-AR molecules after being screened with a threshold binding energy value.

Atenolol, a standard drug molecule used for the treatment of cardiovascular disease, was considered a control molecule to narrow down the molecular space. The binding affinity score of Atenolol was found to be −7.30 and −7.40 Kcal/mol in docking with β1- and β2-AR, respectively. Therefore, the above respective binding affinity score was considered as a threshold value to significantly narrow down the chemical space. By considering the above binding energy as a threshold, it was noticed that most of the docked molecules met the aforementioned criteria. Hence, the target’s threshold value was further modified and defined as −10.00 Kcal/mol, which resulted in the retention of a total of 6611 and 7053 molecules for β1- and β2-AR, respectively. The binding energy of the above molecules was plotted, and it is given in Figure 2. Moreover, the range of binding energy was found to be −2.20 to −12.60 Kcal/mol, and −2.10 to −13.10 Kcal/mol for β1- and β2-AR, respectively.

Further, the ML approach was employed in the present study, which is a very effective and popular technique used to segregate active and inactive molecules from an unknown dataset based on the information of known active and inactive molecular datasets. In this approach, initially, the known active and inactive molecular datasets were collected for the specific target. Herein, molecules retained after the screening through molecular docking for both the β1- and β2-AR targets were considered as the test set for β1- and β2-AR, respectively. A set of active and decoy molecules for both the target receptors were collected from the DUDE database [27]. A total of 458 and 447 active molecules were collected for β1 and β2-AR, respectively. Similarly, a total of 15,958 and 15,255 decoys were collected for β1 and β2-AR, respectively. Amalgamated active and decoys molecules were considered as the training set. Both the training and test molecular datasets for each target receptor were considered for molecular descriptor generation using the PaDEL descriptors tool [28]. Thereafter, six different ML approaches, such as decision tree (DT), random forest (RF), logistic regression (LR), gradient boosting machines (GBM), k-nearest neighbor (kNN), and support vector machine (SVM), were used to segregate the active and inactive molecules. In particular, a total of 1275 significant features were found from 1876 PaDEL descriptors through Wilcoxon’s rank sum test with a significance of *p* < 0.05. The above significant features were used for ML model building. Furthermore, McNemar’s test showed no significant difference between the training and the validated ML model class labels for β1- and β2-AR. A number of performance indices including precision, recall, F-score, accuracy, Matthew’s correlation coefficient (MCC), and confusion matrix (CM) were calculated using six ML models for both β1- and β2-AR and these are given in Table 1.

**Figure 3:**
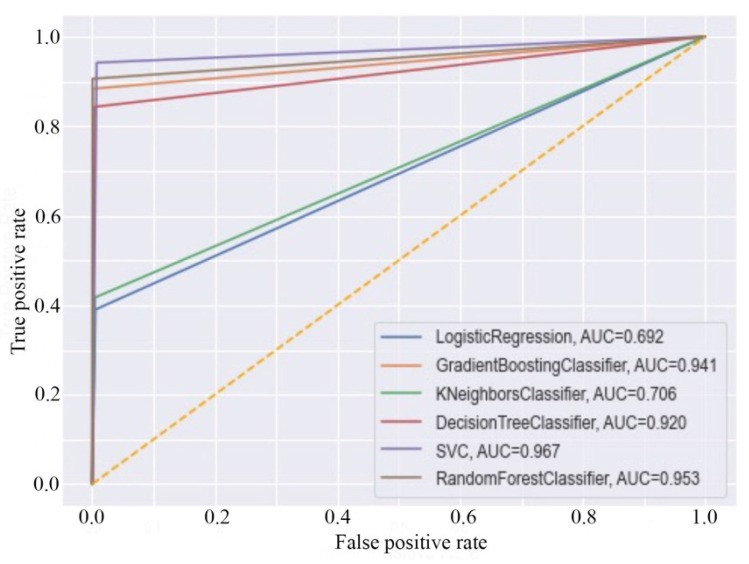
Receiver operating characteristic (ROC) curves of the ML models.

Further, the receiver operating characteristic (ROC) curve of each model was generated, and it is given in Figure 3. In particular, the ROC plot is a representation of the false positive rate (*x*-axis) and the true positive rate (*y*-axis) for all the samples’ thresholds between 0 and 1. The area under curve (AUC) value of SVM, kNN, RF, GBM, LR, and DT was found to be 0.967, 0.706, 0.953, 0.941, 0.692, and 0.920, respectively. The above data undoubtedly explain the significant efficiency of each model.

A cumulative analysis of the molecules from all approaches was performed. The highest number of molecules of at least of two combined approaches were checked and finally a total of 19 and 38 molecules were categorized as active for β1- and β2-AR, respectively. Further, the molecules obtained from the ML analyses were subjected to pharmacokinetic profile evaluation using the SwissADME tool. A number of pharmacokinetic and drug likeness parameters were estimated. Based on the high solubility, good GI absorption, lipophilicity, and synthetic accessibility less than 7 were used to reduce the chemical space for both β1- and β2-AR. Based on the above criteria, it was observed that 4 and 15 molecules for β1- and β2-AR, respectively, failed to pass at least one assigned criterion and therefore were discarded from the list for further analysis.

Molecules remaining after pharmacokinetic analysis were further used for similarity searching against two highly regarded chemical databases viz. DrugBank and ChEMBL. The main objective of the similarity search was to find potential compounds against any existing drug or any standard chemical entity similar to the query molecules. A simplified molecular input line entry system (SMILES) format of 15 and 23 molecules for β1- and β2-AR, respectively, was given as input to the in-house developed Python script, which finds structurally similar molecules based on molecular fingerprints. In the case of β1-AR, from ChEMBL, a total of 12 molecules were found to have a Tanimoto coefficient more than and equal to 0.6. PubChem_26183498 was found to be about 97% similar to CHEMBL4285281. It is also important to note that compound CHEMBL4285281 has already been tested experimentally and its inhibitory activity was found to be 1.7 nM [29]. The second most highly similar PubChem molecule was found to be PubChem_21122992, which showed similarity with CHEMBL4285281 of the ChEMBL database. The inhibitory activity of CHEMBL4285281 was recorded as 17 nM [29]. From the DrugBank database, a total of 774 drugs were found to be similar to the query molecules, having a Tanimoto coefficient of greater than and equal to 0.6. Both PubChem_87666520 and PubChem_153007611 were found to be similar, with Bromocriptine (CHEMBL493) having a Tanimoto coefficient of 0.699 and 0.684, respectively. From the DrugBank similarity search analysis, it was revealed that PubChem_21122992 was found to be highly similar to the compound DB00714, with a Tanimoto coefficient score of 0.947. It is also interesting to observe that PubChem_21122992 was found to be highly similar with the hit compound CHEMBL4285281, having a Tanimoto coefficient score of 0.781. Moreover, compounds PubChem_26183498, PubChem_87666520, and PubChem_15300761 were also found to be highly similar molecules matched against the similarity search with DrugBank compounds, having a Tanimoto coefficient of 0.730, 0.746, and 0.777, respectively. Hence, from the similarity search analyses, PubChem_21122992, PubChem_26183498, PubChem_87666520, and PubChem_153007611 were considered as promising molecules of the β1-AR and subjected to further analysis.

**Figure 4:**
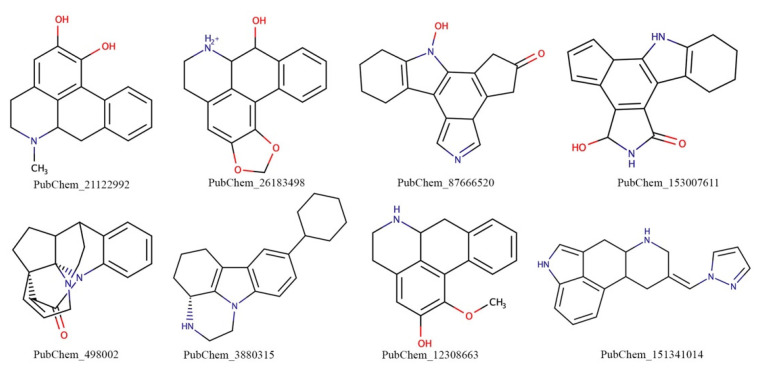
Two-dimensional representation of the final promising molecules for β1- and β2-AR.

In the case of β2-AR, the Tanimoto coefficient cut-off was considered as 0.6 (more than or equal to) and a total of 10 molecules having PubChem IDs 26183498, 46228996, 498002, 152639030, 12308663, 3880315, 3489197, 151341014, 152639030, and 88537601 were found to match the compounds of the DrugBank and ChEMBL databases. Particularly, DrugBank compounds DB01466, DB01200, DB01017, and DB01220 were found to be structurally similar to the above screened out docked molecules. Similarly, the ChEMBL database compounds CHEMBL4285281, CHEMBL493, CHEMBL1082723, CHEMBL421871, and CHEMBL442 were also found to be structurally similar to the query molecules. Further, a detailed literature study was carried out to explore the importance of the above molecules from PubChem, and the corresponding molecules from DrugBank and ChEMBL in connection with cardiovascular diseases. The relevance of cardiovascular disease of the DrugBank and ChEMBL molecules was identified and the most similar docked molecules to those identified molecules were selected for further analysis. Finally, PubChem_498002, PubChem_3880315, PubChem_12308663, and PubChem_151341014 were found to be crucial for cardiovascular diseases and considered to be MD simulation analyses. Two-dimensional representations of the final molecules for both β1- and β2-AR are given in Figure 4. All finalized molecules (Figure 4) consist of a number of important functional groups and aromatic as well non-aromatic cyclic rings. The presence of such functional groups might play important roles in forming crucial binding interactions with amino acid residues at the active site cavity and hence can exhibit the inhibitory/modulatory activity of the studied receptors.

### 2.2. Binding Interaction Analysis

The binding interactions between the final proposed molecules of both β1- and β2-AR were explored through the PLIP web server [30]. Moreover, the binding interactions of Atenolol with both β1- and β2-AR, and the interactions of the respective co-crystal ligand were also explored. The binding energies of all final molecules are given in Table 2.

#### 2.2.1. β1-Adrenergic Receptor

**Figure 5:**
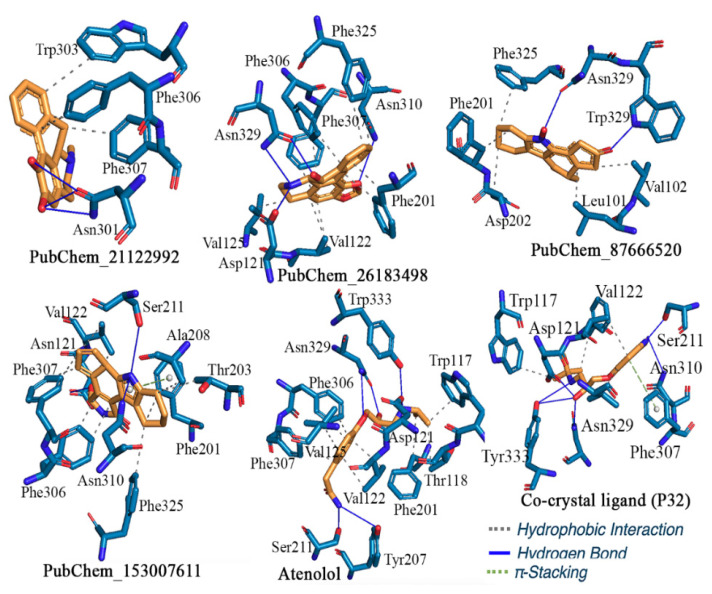
Binding interaction profile of the final molecules for β1-AR, atenolol, and P32.

The binding interaction profile of the final proposed molecules for the target β1-AR along with the control compound Atenolol and co-crystal bound ligand is given in Figure 5. Analysis of the binding interaction profile of PubChem_21122992 revealed that residue Asn310 of β1-AR formed three hydrogen bond (H-bond) interactions with the hydroxyl groups. The phenyl ring without a hydroxyl group present in PubChem_21122992 was found to be important to impart hydrophobicity and formed two hydrophobic interactions with the residue Phe306. Moreover, Trp303 and Phe307 created hydrophobic interactions. A number of potential binding interactions were observed between crucial amino acid residues of β1-AR and the PubChem_26183498. One of the oxygen atoms of the dioxolane ring formed a H-bond with Asn310. The nitrogen atom of the piperidinium ring present in PubChem_26183498 established H-bond interaction with each of the Asp121 and Asn329 residues separately. Another H-bond was also found to form between the hydroxyl group of PubChem_26183498 and residue Asn329. Beyond the above-mentioned interactions, several hydrophobic interactions were also observed between the amino acid residues of β1-AR and atoms of PubChem_26183498. The phenyl ring attached to the dioxolane ring formed one and two hydrophobic interactions with Phe307 and Val122, respectively. The terminal phenyl ring of PubChem_26183498 established several hydrophobic contacts with Phe201, Phe325, and Phe306. The piperidinium ring was also observed to be hydrophobically linked to residue Val122. Another promising compound for β1-AR, PubChem_87666520, was found to establish several H-bonds and hydrophobic interactions with a few important amino acids present at the active site cavity. The hydroxyl group attached to the nitrogen atom and -oxo group connected with the pentacyclic ring in PubChem_87666520 were found to be crucial to the formation of H-bond interactions with Asn329 and Trp330 residues, respectively. The non-aromatic hexa-cyclic ring present in PubChem_87666520 was found to be crucial to the formation of hydrophobic contacts with Asp200, Phe201, and Phe329. Moreover, Leu101 and Val102 participated in the formation of hydrophobic contacts with PubChem_87666520. PubChem_153007611 is a more or less compact structure found to be crucial for β1-AR. A number of binding interactions including hydrogen and hydrophobic contacts along with π-stacking interactions were found between PubChem_153007611 and the active site residues of β1-AR. The hydroxyl group and nitrogen atom present in the penta-cyclic ring of PubChem_153007611 formed two H-bond interactions with residue Asp121. Each of the Ser211 and Asn310 residues formed a H-bond with the nitrogen atom of the pyrrole ring present in PubChem_153007611. Each of the amino acid residues Val122, Phe306, and Phe307 established two hydrophobic contacts with PubChem_153007611. PubChem_153007611 was shown to create hydrophobic contacts with residues Phe201, Thr203, Ala208, and Phe325. Furthermore, the pyrrole ring was observed to be critical for establishing the π-stacking with Phe201. Several essential binding interactions with ligand-binding amino acids at the active site cavity were discovered in both standard compounds: Atenolol and P32. For the control compound Atenolol, the H-bond interactions were discovered to be crucial for amino acid residues Asp121, Tyr207, Ser211, Asn329, and Tyr333. Further, amino acid residues Trp117, Thr118, Val122, Val125, Phe201, Phe306, and Phe307 were found to be critical for the formation of hydrophobic contacts with Atenolol. The co-crystal bound ligand P32 was found to form H-bond interactions with amino acid residues Asp121, Asn310, Asn329, and Tyr333 of β1-AR. Moreover, the Trp117, Val125, Phe201, and Asn310 amino acid residues of β1-AR were found to form hydrophobic interactions with P32. Beyond the above, Phe307 created the π-stacking interaction with P32.

**Figure 6:**
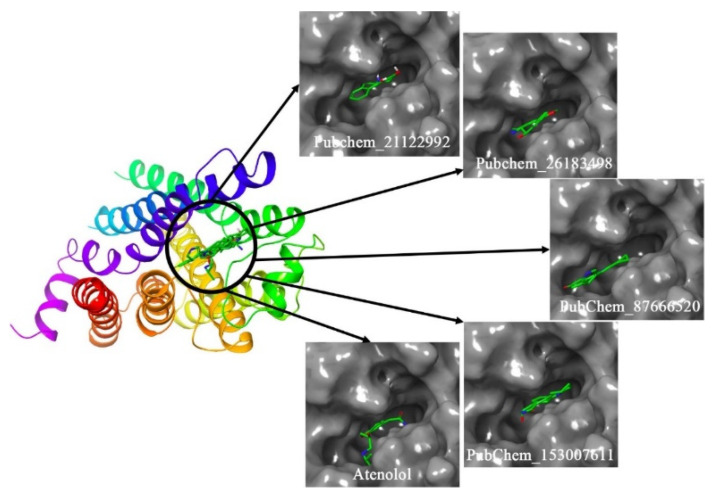
Binding mode of the proposed β1-AR molecules.

The binding mode of the molecules inside the receptor cavity of β1-AR was explored and it is given in Figure 6. It can be seen that all molecules perfectly fitted inside the β1-AR receptor with an optimized orientation to form a maximum number of binding interactions.

Ghabbour et al. [31] synthesized some new oxime ether derivative compounds and performed their molecular docking study against β1-AR. According to the findings of the mentioned study, the key amino acids Phe201, Tyr207, Ser211, Phe216, and Phe307 of β1-AR interacted with the oxime ether derivatives. It is worth noting that, except Phe216, all other amino acids were found to interact with β1-AR in the current study. In another study, a structure-based drug design approach was used for the fragment screening of β1-AR molecules [11]. The potentiality of few important amino acid residues, such as Ser211, Ser215, Ser121, and Asn329, was identified to participate with their studied compounds using the molecular docking study. In the present study, the compounds were also observed to interact with a number of key amino residues, including Ser211, Ser121, and Asn329. As a result of the aforementioned depiction, it can be assumed that the binding interaction profile of the current proposed molecules is consistent with findings from various studies.

#### 2.2.2. β2-Adrenergic Receptor

**Figure 7:**
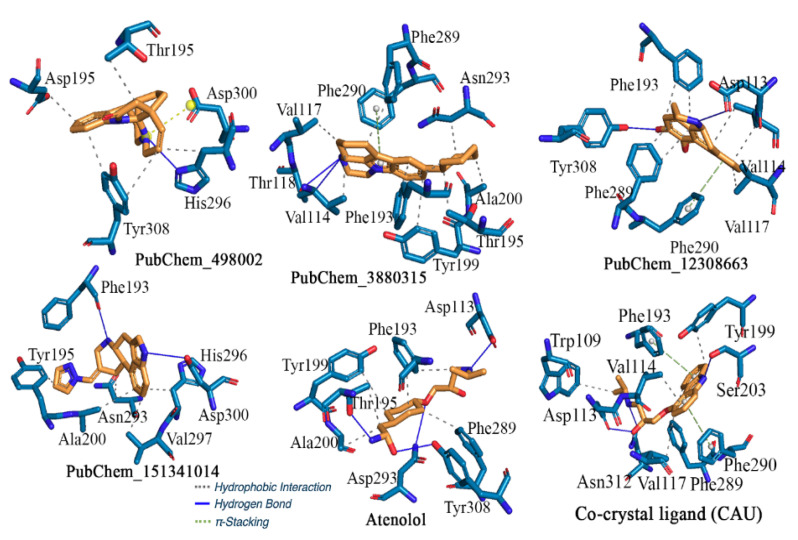
Binding interaction profile of the final molecules for β2-AR, Atenolol, and CAU.

Figure 7 shows the results of the binding interactions profile analysis of the proposed compounds for β2-AR. One of the two nitrogen atoms present in PubChem_498002 was shown to establish a hydrogen bond with residue His296 and a π-stacking interaction with Asp300. The importance of phenyl, terminal hexa-, and pentacyclic rings was discovered in the formation of hydrophobic interactions with residues Asp192, Thr195, His296, and Tyr308 of β2-AR. With some ligand-binding amino acids of β2-AR, PubChem_3880315 generated multiple H-bonds, hydrophobic interactions, and π-stacking, which can be considered as crucial for explicating inhibitory action for β2-AR. Through one H-bond interaction, the amine group present in PubChem_3880315 interacted with each of the residues Val114 and Thr118. The π-stacking interaction was found to participate with Phe290 of β2-AR with the pyrrolidine ring of PubChem_3880315. Except for piperazine, all of the rings present in PubChem_3880315 were found to be essential for the hydrophobic interaction with important β2-AR amino acids, such as Val114, Val117, Phe193, Thr195, Tyr199, Ala200, Phe289, and Asn293. The binding interaction profile of another proposed compound PubChem_12308663 with β2-AR was explored and the intermolecular interactions critically analyzed. The amine and hydroxyl groups of PubChem_12308663 potentially interacted with amino acid residues Asp113 and Tyr308 through H-bond interactions. Val114, Phe193, and Phe289 formed two hydrophobic bond interactions with phenyl rings of PubChem_12308663. Moreover, Val117 and Phe290 were also found to be critical for interaction with one of the phenyl rings of PubChem_12308663 through hydrophobic and π-stacking interactions, respectively. Another promising molecule PubChem_151341014 identified as a prominent modulator inhibitor for β2-AR was also found to form H-bond and hydrophobic interactions with active site amino acid residues of β2-AR. In particular, residues Phe193 and Asp300 were seen to interact with the nitrogen atom of fused hexa-cyclic and penta-cyclic rings, respectively. The presence of a phenyl ring in the molecular system helped to impart the hydrophobicity. The single phenyl ring present in PubChem_151341014 was found to be connected with residues His296, Val297, and Ala200 via hydrophobic contacts. Both the pyrazole and piperidine groups of PubChem_151341014 played an important role in the formation of hydrophobic interactions with Tyr199 and Asn293, respectively. The amine group in Atenolol’s linear chain created an H-bond interaction with residue Asp113. Atenolol’s terminal amine, keto, and oxo groups may have created H-bond interactions with Thr195, Asn293, and Tyr308, respectively. In addition to this, few other residues including Phe193, Thr195, Tyr199, Ala200, and Phe289 of β2-AR formed hydrophobic interactions with Atenolol as observed in the molecular docking. The crystal structure of β2-AR bound with co-crystal ligand (CAU) was also assessed to explore the binding interactions analysis. It was discovered that the essential amino acid residues Asp113, Thr195, Ans293, and Tyr308 created H-bond interactions with CAU. In addition to the above, hydrophobic interactions were also observed between amino acids Phe193, Thr195, Tyr199, Ala200, and Phe289, and CAU.

**Figure 8:**
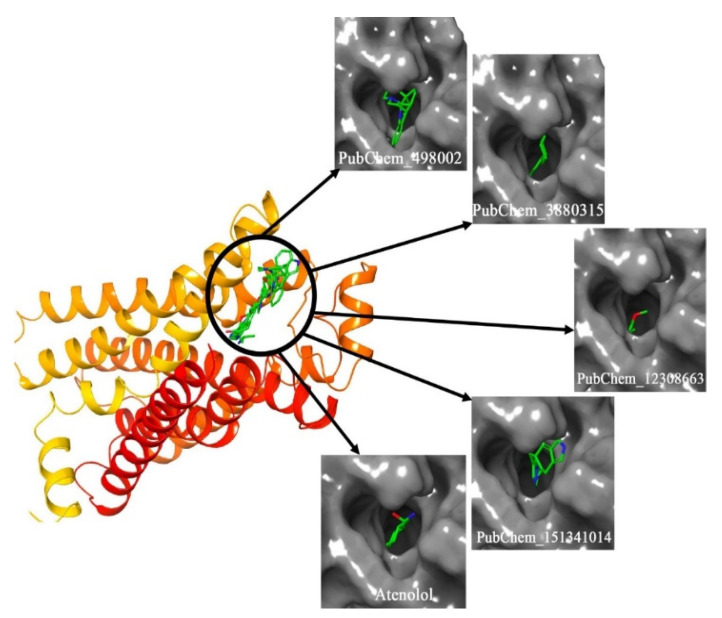
Binding mode of the proposed β2-AR molecules.

The binding modes of each proposed molecule and Atenolol for β2-AR are given in Figure 8. It can be seen that all molecules were buried inside the receptor. It also can be noticed that all molecules fitted in almost the same position.

Bai et al. [32] explored β2-AR ligands through virtual screening and MD simulation analysis. In the said study, the authors performed the virtual screening through MolGridCal and Autodock Vina (ADV) tools. Based on binding energy ranking, they reported three potential molecules for β2-AR. Molecular binding interaction analysis revealed that the top ranked molecules interacted with amino acid residues Asp113, Ser203, Ser207, Asn293, Tyr308, and Asn312. Interestingly, in the current study, except residue Ser207, all other amino acids were also found to interact with the proposed molecules. Another study by Kolb et al. [33] explored structure-based screening of β2-AR molecules using the DOCK program. The authors considered about one million compounds from the ZINC database and utilized the same protein crystal structure from RSCB-PDB (PDB ID:2RH1), as used in the present study. Finally, the said study reported six molecules found to be crucial for inhibiting β2-AR. As per the analyzed data, the binding interaction analysis reported that residues Asp113, Thr195, Ser204, and Tyr308 were important for interaction formation. The proposed molecules in the current study were also found to interact with the above amino acid residues. Yang et al. [34] screened the β2-AR agonist from Fuzi and Chuanwu through the pharmacophore virtual screening approach. At the end, they reported Aconine, Hypaconine, Chasmanine, and Karakolidine as crucial molecules as a β2-AR agonist. The authors found residues Asp113, Asp192, Ser203, Ser207, Lys305, Tyr308, and Asn312 of β2-AR binding-interacting amino acids with the final proposed above molecules. In the current study, a similar binding interaction profile was found. Therefore, from the above observations, it is undoubtedly clear that the binding interaction profile of the proposed molecules for β2-AR was substantiated through the literature.

### 2.3. Pharmacokinetic, Drug-Likeness, and Toxicity Assessment

A number of drug-likeness and pharmacokinetic parameters were calculated for all proposed molecules, and these are given in Table 3. The molecular weight of all proposed molecules was found to be within the range of 267.320 to 294.430 g/mol, which indicated the suitability of penetration through the membrane. It is important to note that molecules possess either zero or one rotatable bond, which, given the rigidity, will help to retain conformational stability in dynamic states. It is illustrated that for a lead-like molecule, the topological surface area (TPSA) should be less than or equal to 140 Å^2^. Not a single molecule was found to have TPSA > 140 Å^2^, suggesting the lead-like behavior of the molecule. The aqueous solubility is one of the important criteria for the absorption of the molecule and is crucial for delivering a sufficient quantity of active ingredient in a small volume. Solubility in the aqueous medium of all the molecules was assessed through LogS and solubility class (SC). All molecules were found to be soluble in nature and LogS higher than −5.00, which clearly explained the absorptivity of the compounds well. High gastrointestinal (GI) absorption of each molecule was suggested to be orally active in nature. Synthetic accessibility (SA) less than 5 strongly indicated that not a single molecule is difficult to synthesis. The bioavailability score (BS) of all compounds was 0.55, which explained the good pharmacokinetic properties [35]. The lipophilicity of any molecule can be examined through the partition coefficient between n-octanol and water (LogP). It is reported that a value of LogP > −6 of any compound is suitable for good absorption. All proposed molecules were found to have a LogP value in the range of 1.90 to 3.35, which substantiated their potential in nature. Hence, the above observations and discussion clearly suggests that all molecules follow a good pharmacokinetic profile and might be lead-like molecules.

In order to check the toxicity of the final molecules, a number of parameters related to the toxicity were calculated and these are given in Table S1 (Supplementary Materials data). All four compounds belonging to β1-AR and PubChem_498002 were found to be non-mutagenic in nature. Moreover, PubChem_3880315, PubChem_12308663, and PubChem_151341014 possibly exhibit mutagenicity to some extent, which suggests further optimization. The maximum tolerated toxic dose for a compound is considered to be low if it is less than 0.477 mg/kg/day [36]. The maximum tolerated toxic dose was found to be 0.05, −0.18, −0.12, 0.09, −0.25, −0.20, −0.01, and −0.88 mg/kg/day for PubChem_21122992, PubChem_26183498, PubChem_8766520, PubChem_153007611, PubChem_498002, PubChem_3880315, PubChem_12308663, and PubChem_151341014, respectively. The above data indicates the acceptability of the molecules in regards to the maximum tolerated toxic. The cardiotoxicity of the molecules was checked through the hERG-I/hERG-II inhibition profile, which is based on the inhibition of potassium channels encoded by hERG (human ether-a-go-go gene). All proposed molecules were found to show no indication of ventricular arrhythmia upon administration. The hepatoxicity of each molecule was explored and found to be negative except for PubChem_151341014, which indicates no disruption of the normal liver function on the intake of these compounds. The skin sensitization of all molecules was revealed as negative, which clearly indicates that there is no potential skin irritation or allergenic effect using these molecules. The oral toxicity (LD50) of all molecules was found to be low (<3.5 mol/kg). The oral chronic toxicity of each molecule was found within the recommended range [37]. Moreover, the other parameters reported in Table S1 also suggested either a non-toxic or low toxic nature of the molecules.

### 2.4. Molecular Dynamics Simulation Analyses

To explore the time-dependent dynamic behavior of any protein-ligand complex, MD simulation is an excellent and widely used computational approach of the scientific community. The MD simulation can provide a detailed conformational change and orientational fluctuation along with intra- and inter-molecular binding interaction stability. Herein, for all the final proposed molecules and along with the standard Atenolol bound with respective targets, β1- and β2-AR were considered for 50 ns MD simulation analyses. After successful completion of the MD simulation run, the numbers of trajectory analysis parameters including the protein-backbone RMSD, ligand RMSD, radius of gyration (RoG), and intermolecular hydrogen bond interactions were calculated and explored. The average, maximum, and minimum values for protein-backbone RMSD, ligand RMSD, and RoG are given in Table S2 (Supplementary Materials data). Each important MD simulation trajectory analysis parameter is discussed subsequently.

#### 2.4.1. Root-Mean Square Deviation

**Figure 9:**
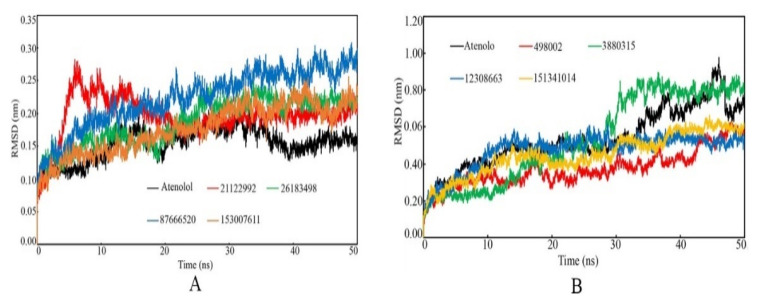
Protein backbone RMSD vs. time of the simulation. (**A**): β1-AR, (**B**): β2-AR.

The protein backbone RMSD calculates the average changes in the displacement of selective atoms for a specific time frame with respect to the backbone of the native structure. This RMSD parameter is quite useful to explore the overall stability of the bio-molecular system (e.g., protein-ligand) in a dynamic environment. It is illustrated that low deviation of RMSD values throughout the simulation time span indicates higher stability of the molecular system. With a similar postulation, the conformational and orientational deviation of the small molecules inside the active site cavity during the simulation is indicated by the ligand RMSD. High fluctuation of the ligand RMSD may suggest more conformational and rotational alteration in the dynamic states. In the current study, the time-dependent β1- and β2-AR backbone RMSD value of each frame was extracted and it is plotted in Figure 9. It can be seen that except for the β1-AR backbone bound with PubChem_21122992 (Figure 9A), all other complexes remained consistent throughout the simulation run period. The β1-AR backbone bound with PubChem_21122992 initially deviated at a higher value and later at ~15 ns, the simulation system gradually achieved its consistency. Although the β1-AR backbone bound with Atenolol was found to stabilize with lower RMSD compared to others, not a single backbone bound with the proposed molecules was found to deviate with an extremely high value as it always remains below 0.30 nm.

The RMSD of the β2-AR backbone bound with the final proposed molecules and standard compound Atenolol was calculated and it is given in Figure 9B. It was observed that the RMSD of β2-AR backbone for all ligand-bound complexes oscillated within the range of 0 to 0.977 nm. It is also worth noting that the β2-AR backbone bound with standard compound Atenolol was found to deviate more frequently than the other proposed compounds. Such steady deviation of the β1- and β2-AR backbone bound with proposed molecules explained the stability of the protein-ligand complex in dynamic states.

**Figure 10:**
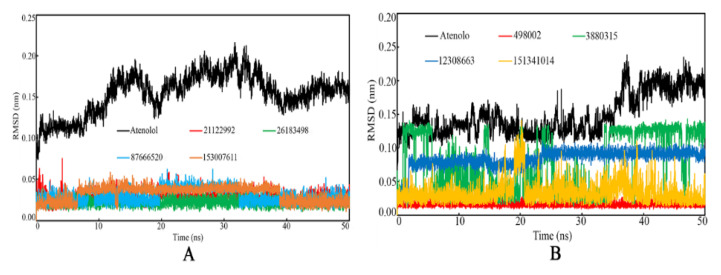
Small molecule backbone RMSD vs. time of the simulation. (**A**): Complex with β1-AR, (**B**): Complex with β2-AR.

The deviation of the individual proposed compound during the MD simulation was also explored through evaluation of the ligand RMSD values for all the molecules of β1- and β2-AR along with Atenolol. The ligand RMSD values were plotted against the time of simulation and are given in Figure 10. The RMSD of standard compound Atenolol bound with both β1- and β2-AR was found to deviate with a higher value in comparison to the proposed molecules. Such an observation indicates that the standard compound Atenolol might have undergone some degree of conformational changes at the active side of both the β1- and β2-AR, which resulted in higher RMSD values. However, all proposed ligands bound with β1-AR were shown to have relatively lower RMSD values and deviation of the RMSDs ranging from 0 to 0.075 nm. On the other hand, for ligands bound with β2-AR, the RMSDs were seen to deviate a little bit higher, except for the compounds PubChem_49008 and PubChem_151341014. Despite the fact that the molecule PubChem_12308663 had a larger RMSD, the magnitude or range of deviation observed was quite small. PubChem_3880315 was noticed to alter its conformational orientation regularly, which might result in variation in the RMSD value during simulation.

#### 2.4.2. Radius of Gyration

**Figure 11:**
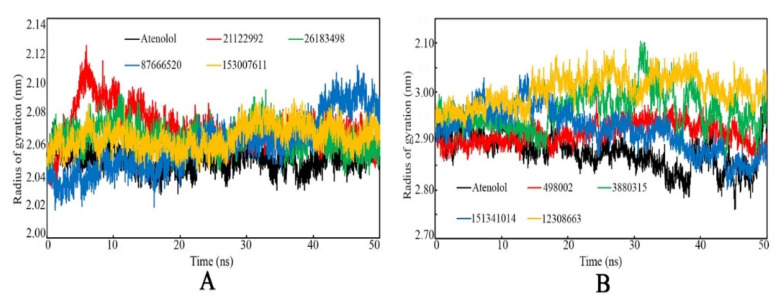
Radius of gyration vs. time of the simulation. (**A**): β1-AR, (**B**): β2-AR.

The rigidity and compactness of the protein-ligand systems can be analyzed through RoG values, which were explored from the MD simulation trajectories. It is the mass-weighted RMS distance of a collection of atoms from their common center of mass [38]. It is postulated that RoG is a crucial MD simulation parameter to observe the overall dimensions and the change in the initial protein structure. Hence, the protein rigidity and folding changes can be assessed using the RoG analysis. The RoG value of both the β1- and β2-AR bound with the respective proposed molecules and Atenolol was calculated and is plotted in Figure 11. A consistent RoG value was observed for both the studied targets, which can clearly explain the steadily folding nature of proteins and/or most tightly packed protein of the system in the dynamic states.

#### 2.4.3. Hydrogen Bonding Interaction Analyses

**Figure 12:**
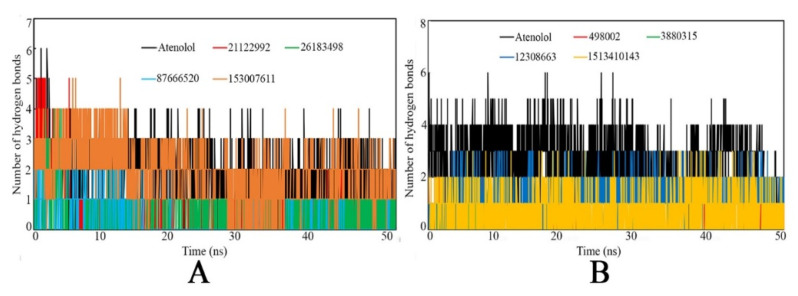
Number of hydrogen bonds vs. time of the simulation. (**A**): Complex with β1-AR, (**B**): Complex with β2-AR.

The distance between the H-bond acceptor and H-bond donor atoms of the counter portion of the ligand and protein/receptor influences the formation of intermolecular H-bonds. Moreover, the H-bond interaction helps to stabilize the protein-ligand complex system, which is highly important and relevant to any bio-molecular system for assessing their interaction integrity. After MD simulation completion, each simulation trajectory was utilized to compute the number of H-bond interactions present in each frame, and is presented in Figure 12. Particularly, the MD simulation run was used to calculate the presence of the number of H-bonds between the studied ligands and with their respective targets in each frame. It was observed that all of the frames either formed no interactions or a maximum of six H-bond interactions.

For all identified proposed compounds, the overall distribution of H-bond interactions determined over the course of the simulation run was found to be different for many frames (high and low H-bonds), which might be due to the conformational changes of each compound along with the distance between the H-bond acceptor and counter H-bond donor atoms possessed within or out of the range. In particular, for the β1-AR protein, compounds Atenolol, PubChem_21122992, and PubChem_153007611 showed relatively higher numbers of H-bond formation during the initial phase of the simulation span. On the other hand, for the β2-AR protein, the compounds Atenolol and PubChem_151341014 were attributed to relatively higher numbers of H-bond interactions than the other compounds. Therefore, certainly, it might be possible that the presence of H-bonds between putative ligands and associated receptors contributes to maintaining the protein-ligand complex’s dynamic stability.

#### 2.4.4. Post-MD Simulation Binding Interaction Analysis

After the MD simulation run was completed, the protein-ligand complex for all proposed compounds for both target receptors was extracted. The binding interactions between the respective studied proteins and proposed ligands were compared to those before the simulation began. Figures S2 and S3 (Supplementary Materials data) show the binding interaction profiles of β1- and β2-AR post-MD simulation. According to the results obtained from the comprehensive analysis of post-MD extracted complexes, all molecules were able to retain a few common binding contacts or remained in close proximity to the ligand-binding amino acids as discovered before the MD simulation. In addition, a number of novel binding interactions with amino acids were also discovered. The formation of new binding contacts and the breaking of old interactions/contacts could be attributed to the conformational changes in molecules inside the pocket during simulation. Particularly, compound PubChem_26183498 was found to retain a similar binding interaction profile with amino acid Asn310 and Phe201 of β1-AR as the pre-simulation state. In both situations, before and after the MD simulation, one of the same amino acid residues Phe201 of β1-AR was identified to interact with compound PubChem_87666520. Similarly, PubChem_153007611 critically retained binding interactions with several residues, namely Asp121, Val122, Ala208, and Asn310, after MD simulation. However, an interesting finding was observed for PubChem_21122992, which failed to maintain any common binding interactions with β1-AR on the pre- and post-MD simulation. Intriguingly, PubChem_21122992 was shown to create multiple binding contacts with amino residues near the ligand-binding amino acids observed in the pre-simulated state of the complex. In the pre- and post-MD simulations, Atenolol bound to β1-AR was observed to retain binding contacts with Asp121 and Phe306.

Post-MD simulation retrieved the binding interaction profile for PubChem_498002 and revealed that Tyr308 successfully reserved the binding interaction with β2-AR, before and after the simulation ended. Residues Val114, Thr195, Ala200, and Phe289 were found to be important to keep the binding interactions attributed by hydrogen bonds or hydrophobic interactions with PubChem_3880315 in both the pre- and post-MD simulation state. In the molecular docking complex or pre-MD simulation state and post-MD simulation complex, PubChem_12308663 was observed to reserve the common binding contacts with residues Val117, Phe193, and Phe289. After MD simulation, PubChem_151341014 bound with β2-AR was discovered to have similar binding interactions with residues Tyr199, Ala200, and Val297. In the pre- and post-MD simulation states, standard compound Atenolol coupled with β2-AR successfully conserved its similar binding interactions profile with residues Phe289 and Tyr300. Following the MD simulation, it was revealed that all molecules including the standard Atenolol remained inside the receptor cavity, which clearly explained the molecules’ high binding affinity towards the studied receptors. Furthermore, it was obvious that few molecules had broken some existing interactions and established new contacts in order to maintain their conformational integrity within the receptor cavity.

It is also crucial to notice the orientation of the lipid bilayer and protein molecule along with their position of the correspondingly bound small molecules to each receptor. Due to conformation analysis during the MD simulation, the position of the ligand and structural orientation of the protein and lipid bilayer may be altered. There was no such evidence that small molecules have a lower binding affinity towards the receptor, implying that the identified small molecules have no possibility of coming out of the receptor cavity. To explore the above possible events, the protein-ligand complex of both β1- and β2-AR buried inside the lipid bilayer was extracted and is depicted in Figures S4 and S5 (Supplementary Materials data). From both Figures S2 and S3, it was clearly seen that there are no noticeable positional changes in the lipid, ligand, and protein except a few conformational alterations. Therefore, it can be concluded that all proposed small molecules remained stable inside both the β1- and β2-AR active pockets, in the presence of the lipid bilayer membrane in the dynamic states.

## 3. Materials and Methods

Virtual screening (VS) is an important and efficient computational approach for retrieving active chemical compounds from a pool of large chemical databases against specific bio-molecular receptor/protein targets. The technique has become very popular for drug discovery research among academics and/or pharmaceutical companies all around the world [39]. Due to its excellent power to screen the effective and potential molecules prior to synthesis, the VS technique has become the favorite choice of the drug discovery community. It is vital to note that the physical presence of the investigated chemicals is not necessary for the execution of the VS technique. Potential molecules can be screened prior to synthesis and thereafter it is only required for synthesis if the VS demonstrates any molecule that possesses good binding potency for the target [40]. In general practice, there are two types of VS, namely the ligand-based VS (LBVS) and the structure-based VS (SBVS). LBVS refers to the screening of chemical databases through any *in-silico* model developed using a set of small molecules, such as the quantitative structure–activity relationship (QSAR) [41] or pharmacophore model [42]. Specifically, LBVS analyzes known bioactive molecules to find structurally varied chemical compounds with similar bioactivity profiles to the query data [43,44]. Most of the LBVS strategies are based on the assumption that a similar structure possesses similar characteristics, which makes it sometimes difficult to find novel chemotypes [45,46]. On the other hand, the SBVS technique comprises the three-dimensional (3D) chemical structure of the target or receptor of interest where molecules belong to the chemical databases are docked differently to predict their best inter-molecular binding orientation for the production of maximum therapeutic effect. Such a technique considers two important factors, such as steric and energetic complementarity between the small molecule and binding site of the target followed by screening of the molecules based on molecular recognition events, such as molecular interactions, binding energetics, and even induce-fit behavior [47,48,49]. The molecular docking-based VS (DBVS) is one of the widely used SBVS strategies in which the active binding mode and binding affinity of the molecules towards the target are estimated [50,51]. Several successful applications of DBVS have already been reported recently in the literature [52,53,54,55,56,57,58]. With the help of the beneficial effects of DBVS, the current study was considered to screen the PubChem database against β1- and β2-AR through the DBVS, ML approach, in silico ADME evaluation, and binding interactions stability assessment through MD simulation studies. The credential of the work has been substantiated by reporting four potential small molecules for β1- and β2-AR.

### 3.1. Compound Dataset Collection and Curation

The entire PubChem chemical dataset, which contains roughly about 99 million small molecules, was downloaded in October 2020. PubChem is one of the largest public repositories of chemical compounds and is widely used in diverse research areas including cheminformatics, chemical biology, medicinal chemistry, and drug discovery [59,60,61,62]. Prior to the use of the downloaded molecular dataset in the SBVS, the entire dataset was curated using a number of parameters including LoF [63], Ghose’s rule [64], Veber’s rule [65], Rule of three [66], and drug-likeness [67]. Overall, by applying the above-mentioned rules, a number of screening criteria were included to sort out the molecules, such as molecular weight < 300, logP <= 5, hydrogen bond (HB) acceptor <= 10, HB donor <= 5, atom counts between 20 and 70, molar refractivity in the range of 40 to 130, number of rotatable bonds <= 10, and total polar surface area <= 140. The RDKit [68] based Python code was developed in house to implement the above criteria and used for the reduction of the chemical space. After successful execution of the Python code, a total of 475,369 molecules were retained. All these retained molecules were further converted into the .pdbqt format using OpenBabel [69], which is a molecular file format conversion tool. Finally, using the vina_split package of ADV [70], all molecules were separated into an individual file for the molecular docking study.

### 3.2. Protein Preparation

The Research Collaboratory for Structural Bioinformatics—Protein Data Bank (RCSB-PDB) [71] is the first open-access digital largest resource of three-dimensional (3D) crystal structures of macromolecules and associated small molecules [72]. The wider community belonging to the scientific disciplines benefit from RCSB-PDB resources by obtaining the 3-D coordinates of macromolecules for computational drug discovery approaches including DBVS. For the current study, the crystal structures of β1- and β2-AR were obtained from RCSB-PDB having PDB IDs 2VT4 [13] and 2RH1 [73], respectively. A number of parameters including the atomic resolution, R-value, and date of deposition in the PDB database were considered to select the crystal structure of β1- and β2-AR. The resolution and R-value of β1- and β2-AR were found to be 2.70 Å and 0.268, and 2.40 Å and 0.232, respectively. The number of amino acid residues was found to be 313 and 500 in β1- and β2-AR, respectively. Two different inhibitor compounds P32 (4-{ [(2S)-3-(tert-butylamino)-2-hydroxypropyl]oxy}-3H-indole-2-carbonitrile) and CAU ((2S)-1-(9H-Carbazol-4-yloxy)-3-(isopropylamino)propan-2-ol) are bound as co-crystal ligand to the β1- and β2-AR, respectively. Each of the receptors was prepared using the Autodock tools (AD4)]. All the hetero atoms including water molecules were deleted. The missing atoms and residues were checked and repaired. The polar hydrogens and Gasteiger charges were added. The atoms of the molecules were assigned as AD4 type and saved as .pdbqt format for further use as the input in ADV.

### 3.3. Molecular Docking

Molecular docking is an excellent and powerful approach to screen a large number of chemical compounds for a specific target. In the current study, two targets, such as β1- and β2-AR, were considered for molecular docking with PubChem compounds using ADV [70]. ADV is a freely available open-source molecular docking tool and has been highly cited since its availability from 2010 [74]. ADV is an excellent choice as a widely used docking tool to determine accurate and rapid binding affinity prediction between the protein and ligand [75]. The empirical scoring function is used by ADV and also comprises the Gaussian steric interactions, repulsion, hydrogen bonds, and hydrophobic and torsion terms [76]. It was illustrated that ADV was developed for parallel computing capability and to predict the binding affinity with better accuracy based on the CASF-2013 benchmark [77]. Along with the prediction of the binding affinity of small molecules, ADV was also found to be excellent for other bio-molecular targets including peptides, proteins, and genes.

Prior to the execution of molecular docking with all chemical compounds, validation of the employed docking protocol is an essential and required step to select accurate parameters. Self-docking is one of the approaches in which the co-crystal bound ligand is re-drawn and docked at the same site where the co-crystal ligand was originally bound [78]. The best docked pose is superimposed to the co-crystal ligand and the RMSD is calculated. It is reported that RMSD < 2 Å successfully validates the molecular docking protocol [79]. Co-crystal-bound ligands in both β1- and β2-AR were re-drawn and docked in the respective target sites. The best docked pose of each target was superimposed with the co-crystal-bound ligand and RMSD was calculated. For both the targets, the active site was considered to be the position where the co-crystal bound ligand was originally present. The grid was optimized by increasing or decreasing the size of the grid box and re-docked with the co-crystal ligand. The size in which the best binding affinity was found was considered as the grid size for docking with the database-filtered molecules. Hence, the grid coordinate for β1- and β2-AR was selected to be (26.617, 4.052, 0.847 Å) and (−35.184, 6.350 and 7.988 Å), respectively, along with the X, Y, and Z coordinates. The optimized grid dimension was considered to be 60 × 60 × 60 Å and 80 × 80 × 80 Å for β1- and β2-AR, respectively.

### 3.4. Virtual Screening

The entire curated PubChem dataset was docked into the active site of both β1- and β2-AR targets using the ADV implemented in Python script. The molecular docking study was performed in the Lamda function of the AWS server. It is a powerful application of Amazon Web Services that runs as an event-driven, serverless platform. In general, it runs the code in response to events and automatically accomplishes the computing resources required by that code. The entire set of curated molecules was docked into both the β1- and β2-AR targets and the binding energy of each molecule was explored. The standard drug, Atenolol [80], was also incorporated in the molecular docking dataset and docked with the same parameters as the PubChem dataset docking for both the β1- and β2-AR targets.

#### 3.4.1. Binding Affinity-Based Screening

Initially, the binding affinity score of Atenolol was checked and considered the same as a threshold value for screening out database molecules with comparatively lower binding energy than Atenolol. Initially, it was found that most of the molecules that were docked had a binding affinity score within the defined threshold. Hence, consideration of the threshold value was increased gradually in order to reduce the chemical space. The molecules obtained after screening through the considered threshold of −10.00 Kcal/mol were used for the ML approach to identify active and inactive compounds. The remaining molecules in the above approach were considered for assessment through pharmacokinetic analysis. Finally, the similarity search of DrugBank [81,82] and ChEMBL [83,84] databases were carried out to retrieve the potential compounds with the most similar chemical components after pharmacokinetic assessment.

#### 3.4.2. Machine Learning Approach

The ML approach is an emerging field of artificial intelligence (AI) and has established significant contributions to drug discovery research [85]. This approach has already been applied in different drug discovery methodologies including molecular property and activity prediction [86,87,88], virtual screening [89,90], retrosynthetic analysis [91,92], and *de novo* drug design [93,94,95]. In the present study, to segregate the active and inactive molecules from the docked dataset, chemical descriptor-based classification was carried out through six different supervised ML models including DT [96], RF [97], LR [98], GBM [99], kNN [100], and SVM [101]. The active and decoy sets for both the β1- and β2-AR targets were retrieved from the DUDE database [27]. Both the active and decoy datasets were considered as the training set and the docked PubChem compounds after screening based on the user-defined binding energy were considered as the test set. Molecular descriptors (2-D and 3-D) and fingerprints of both the training and test set molecules were generated using the PaDEL descriptors generation tool [28]. PaDEL is a publicly available software tool to calculate molecular descriptors and fingerprints for a given set of small molecules. More precisely, the descriptors and fingerprints are calculated using “The Chemistry Development Kit”, such as atom type electrotopological state descriptors, Crippen’s logP and MR, extended topochemical atom (ETA) descriptors, McGowan volume, molecular linear free energy relation descriptors, ring counts, count of chemical substructures identified by Laggner, and binary fingerprints and count of chemical substructures. Initially, Wilcoxon’s rank-sum test was performed to identify the statistically significant (*p* < 0.05) features between active and inactive compounds. These significant features were used to train the ML models using the scikit-learn package in Python3 [102]. *k*-fold cross-validation was used to estimate the skill of the model on *k* different train and test splits. Ten-fold cross-validation was performed to optimize the hyperparameters for all these employed models. In addition, McNemar’s test was performed to identify a statistically significant difference or disagreement between the train and validated ML model class labels with significance *p* < 0.05. Based on true positive (TP), true negative (TN), false positive (FP), and false negative (FN) data, a number of performance indices including precision, recall, F-score, accuracy, MCC, and CM were calculated using various ML models. The following expressions were used to calculate the above-mentioned indices:

(1) $$Precision=\frac{TP}{\left(FP+TP\right)}$$

(2) $$Recall\;\left(or\;Sensitivity\right)=\frac{TP}{\left(TP+FN\right)}$$

(3) $$F-score=\;2\times \frac{TP}{\left(2\times TP+FP+FN\right)}$$

(4) $$Accuracy=\frac{\left(TP+TN\right)}{\left(TP+TN+FP+FN\right)}$$

(5) $$MCC=\frac{\left[\left(TP\times TN)-(FP\times FN\right)\right]}{\sqrt{\left[\left(TP+FN)\times (TP+FP)\times (TN+FP)\times (TN+FN\right)\right]}}$$

(6) $$Confusion\;matrix\;(CM)=\left[\begin{array}{l} TP\;\;FP\\ FN\;\;TN \end{array}\right]$$

Further, predictions of the compounds were made based on their predicted active or inactive nature using the trained models on the test dataset. The contingency table for all the ML models was built, and the active compounds predicted using the majority voting of the ML models (3 and above) were chosen for further evaluation and additional assessments.

#### 3.4.3. *In Silico* Pharmacokinetic Analysis and Toxicity Assessment

In silico pharmacokinetic assessment is one of the important approaches that helps in screening out potential molecules with their better drug-likeness and other chemical safety profiles from a large chemical dataset. Molecules that remained after the ML analysis were considered for the SwissADME tool [103] to calculate a number of pharmacokinetic and drug-likeness properties. The SwissADME, a web-server-based open-source tool, was used for ADME profile prediction for all compounds. Among the several parameters, solubility, human GI, synthetic accessibility, etc. were considered to reduce the chemical space.

The toxicity of the final molecules for both β1- and β1-AR was calculated through the ‘pkCSM’, which is a publicly available web server and is widely used by the scientific community [104]. It is based on graph signatures, for example, toxicity assessment is carried out using the mathematical illustration of any given compound. A number of toxicity parameters including AMES toxicity, maximum tolerated dose (human), hERG-I/hERG-II inhibitor, oral rat acute toxicity, oral rat chronic toxicity (LOAEL), hepatotoxicity, skin sensitization, T. Pyriformis toxicity, and Minnow toxicity, are generated for a given input compound.

#### 3.4.4. Similarity Search of DrugBank and ChEMBL

Similarity search of any query molecule against the target molecular database is an important and well-established virtual screening approach [105,106,107]. The concept of similarity search relies on the basic concept that structurally similar molecules tend to show similar biological activity. In this method, fingerprint-based similarity search of the query molecules based on an extended connectivity fingerprint, up to four bonds (ECFP4), was explored in the small molecular databases followed by the ranked hit molecules according to the Tanimoto coefficient [108,109]. Molecules for both the β1- and β2-AR targets that followed the acceptable pharmacokinetic assessment were considered for the RDKit-based python script as similar molecules from the DrugBank and ChEMBL databases. It takes the SMILES representation of molecules as input and searches the DrugBank and ChEMBL databases with the Tanimoto coefficient [108,109]. The initial hits were arranged according to the ascending order of the Tanimoto coefficient. The molecules that had a Tanimoto coefficient greater than or equal to 0.6 were collected. A detailed study of the target molecules was explored including the experimental biological activity, the target of the molecule, and most importantly the similarity score. Based on the above screening criterion and obtained data, finally, four promising molecules for each of the β1- and β2-AR targets were selected.

### 3.5. Molecular Dynamics Simulation

Molecular dynamics simulation is the approach to explore the behavior and stability of the protein-ligand complex in dynamic states. A 50 ns time span of MD simulation of each protein-ligand complex in the 1-palmitoyl-2-oleoyl-sn-glycero-3-phosphocholine (POPC) lipid bilayer was performed in Gromacs 2021.3 [110]. To prepare the system, each complex was uploaded in the membrane builder online webserver CHARMM-GUI [111]. A total of 187 POPC lipids in both the upper and lower leaflets were added. The system was solvated using the TIP3P [112] water model and neutralized by addition of the required number of Na^+^ and Cl^−^ ions [113]. The CHARMM36 protein force-field and GAFF2 [114] force fields were used to generate the topology of the protein and ligand, respectively. After successful generation of the systems through Membrane Builder of CHARMM-GUI, each system was equilibrated with six short equilibrations of 25 to 100 ps. The protein backbone and side chains along with ions were controlled using the harmonic restraint. Further, harmonic restraint was also applied to the water molecules to restrict them from entering the hydrophobic region of the membrane. The above restraints were slowly reduced in the successive equilibration steps. In the equilibrations, two ensembles were used, such as NVT (constant volume and temperature) followed by NPT (constant pressure and temperature). Followed by the equilibrations, production was carried out for a 50 ns time span with a 2 fs timestep. No harmonic restraints were used during the production stage. After successful completion of the production, a number of parameters including the protein backbone and ligand RMSD, RoG, and hydrogen bond analysis during MD simulation were calculated and analyzed.

## 4. Conclusions

Structure-based virtual screening of the PubChem database followed by ML and similarity-based searching along with MD simulation were carried out to identify potential β1- and β2-AR ligands for therapeutic applications in cardiovascular diseases. Finally, four molecules for each of β1- and β2-AR were found to be promising modulators. High negative binding free energy in molecular docking in comparison to the standard drug Atenolol explained the strong affinity towards the respective target. The binding interaction profile of each molecule was explored, and a number of critical amino acids were found to form hydrogen bonds and hydrophobic interactions. In silico pharmacokinetic analyses revealed that each molecule is highly absorbable in GI, soluble in nature, and not difficult to synthesis. Drug-likeness assessment was explored and all molecules were found to possess lead-like characteristics. The dynamic behavior of the molecules inside the protein cavity was explored through MD simulation. A number of statistical parameters from the MD simulation clearly explained the stability of the protein-ligand complex in dynamic states. Hence, all together, it can be postulated that the proposed molecules through an advanced level of computational drug discovery analyses can be potential modulators for β1- and β2-AR, subjected to experimental validation.

## Figures and Tables

**Table 1:** ML model performance indices for β1- and β2-AR.

Classifier	β1-AR					
	Precision	Recall	F-Score	Accuracy	MCC	CM
SVM	0.92	0.89	0.90	0.89	0.80	TP:325,FP:20,FN:10,TN:5987
RF	0.99	0.71	0.79	0.71	0.64	TP:341,FP:4,FN:140,TN:5857
KNN	0.78	0.57	0.61	0.57	0.29	TP:270,FP:75,FN:198,TN:5799
GBM	0.93	0.87	0.89	0.87	0.79	TP:341,FP:4,FN:40,TN:5957
DT	0.87	0.91	0.89	0.91	0.77	TP:301,FP:44,FN:30,TN:5957
LR	0.86	0.73	0.78	0.73	0.57	TP:297,FP:48,FN:109,TN:5888
β2-AR						
SVM	0.97	0.87	0.91	0.87	0.89	TP:433,FP:14,FN:14,TN:5240
RF	0.99	0.81	0.88	0.81	0.78	TP:447,FP:0,FN:0,TN:15254
kNN	0.87	0.8	0.83	0.80	0.67	TP:390,FP:97,FN:98,TN:15156
GBM	0.97	0.83	0.89	0.83	0.82	TP:447,FP:0,FN:2,TN:15252
DT	0.95	0.97	0.96	0.97	0.93	TP:447,FP:0,FN:0,TN:15254
LR	0.87	0.73	0.78	0.73	0.58	TP:390,FP:57,FN:0,TN:15114

RF: Random Forest; SVM: Support Vector Machine; kNN: k-Nearest Neighbors; GBM: Gradient Boosting Machine; LR: Logistic Regression; DL: Deep learning; TP: True positive; TN: True negative; FP: False positive; FN: False negative.

**Table 2:** Binding energy of the final proposed molecules of β1- and β2-AR.

Molecule		Binding Energy (Kcal/mol)
β1-AR	PubChem_21122992	−11.90
	PubChem_26183498	−11.10
	PubChem_87666520	−10.40
	PubChem_153007611	−12.80
	Atenolol	−7.30
	P32	−8.60
β2-AR	PubChem_498002	−12.20
	PubChem_3880315	−10.70
	PubChem_12308663	−11.10
	PubChem_151341014	−11.80
	Atenolol	−7.40
	CAU	−7.50

**Table 3:** Pharmacokinetic and drug-likeness parameters of β1-AR and β2-AR molecules.

	β1-AR				β2-AR			
Parameters	M1	M2	M3	M4	M5	M6	M7	M8
Formula	C_17_H_17_NO_2_	C_17_H_16_NO_3_	C_17_H_16_N_2_O_2_	C_17_H_16_N_2_O_2_	C_19_H_20_N_2_O	C_20_H_26_N_2_	C_17_H_17_NO_2_	C_18_H_18_N_4_
^1^ MW(g/mol)	267.320	282.31	280.320	280.320	292.370	294.430	267.320	290.36
^2^ NHN	20	21	21	21	22	22	20	22
^3^ NAHA	12	12	5	5	6	9	12	14
^4^ NRB	0	0	0	0	0	1	1	1
^5^ TPSA	43.700	55.300	54.590	65.120	23.550	16.960	41.490	45.640
LogS	−3.39	−3.10	−1.89	−2.38	−3.24	−4.69	−3.54	−3.11
^6^ SC	Soluble	Soluble	Very soluble	Soluble	Soluble	Moderately soluble	Soluble	Soluble
^7^ GI	High	High	High	High	High	High	High	High
^8^ vLoF	0	0	0	0	0	0	0	0
^9^ BS	0.55	0.55	0.55	0.55	0.55	0.55	0.55	0.55
^10^ SA	3.22	3.68	4.21	4.31	4.98	3.51	3.24	3.56
LogP	2.61	2.48	2.20	1.90	2.76	3.35	2.56	2.48

M1: PubChem_21122992; M2: PubChem_26183498; M3: PubChem_8766520; M4: PubChem_153007611; M5: PubChem_498002; M6: PubChem_3880315; M7: PubChem_12308663 and M8: PubChem_151341014; ^1^ Molecular weight; ^2^ Number of heavy atoms; ^3^ Number of aromatic heavy atoms; ^4^ Number of rotatable bonds; ^5^ Total polar surface area; ^6^ Solubility class; ^7^ Gastrointestine absorption; ^8^ Violation of LoF; ^9^ Bioavailability score; ^10^ Synthetic accessibility.

## Supplementary Materials

The following are available online at https://www.mdpi.com/article/10.3390/ijms222011191/s1.

## References

[1] Yang D., Zhou Q., Labroska V., Qin S., Darbalaei S., Wu Y., Yuliantie E., Xie L., Tao H., Cheng J. (2021). G protein-coupled receptors: Structure-and function-based drug discovery. Signal Transduct. Target. Ther..

[2] Salon J.A., Lodowski D.T., Palczewski K. (2011). The significance of G protein-coupled receptor crystallography for drug discovery. Pharmacol. Rev..

[3] Ferguson S.S.G., Barak L.S., Zhang J., Caron M.G. (1996). G-protein-coupled receptor regulation: Role of G-protein-coupled receptor kinases and arrestins. Can. J. Physiol. Pharmacol..

[4] Premont R.T., Gainetdinov R.R. (2007). Physiological roles of G protein-coupled receptor kinases and arrestins. Annu. Rev. Physiol..

[5] Ribas C., Penela P., Murga C., Salcedo A., García-Hoz C., Jurado-Pueyo M., Aymerich I., Mayor F. (2007). The G protein-coupled receptor kinase (GRK) interactome: Role of GRKs in GPCR regulation and signaling. Biochim. Biophys. Acta Biomembr..

[6] Gurevich V.V., Gurevich E.V. (2019). GPCR signaling regulation: The role of GRKs and arrestins. Front. Pharmacol..

[7] Ferguson S.S.G. (2001). Evolving concepts in G protein-coupled receptor endocytosis: The role in receptor desensitization and signaling. Pharmacol. Rev..

[8] Salazar N.C., Chen J., Rockman H.A. (2007). Cardiac GPCRs: GPCR signaling in healthy and failing hearts. Biochim. Biophys. Acta Biomembr..

[9] Rengo G., Perrone-Filardi P., Femminella G.D., Liccardo D., Zincarelli C., De Lucia C., Pagano G., Marsico F., Lymperopoulos A., Leosco D. (2012). Targeting the β-adrenergic receptor system through g-protein-coupled receptor kinase 2: A new paradigm for therapy and prognostic evaluation in heart failure from bench to bedside giuseppe rengo pasquale perrone-filardi. Circ. Heart Fail..

[10] Grahl A., Abiko L.A., Isogai S., Sharpe T., Grzesiek S. (2020). A high-resolution description of β1-adrenergic receptor functional dynamics and allosteric coupling from backbone NMR. Nat. Commun..

[11] Christopher J.A., Brown J., Doré A.S., Errey J.C., Koglin M., Marshall F.H., Myszka D.G., Rich R.L., Tate C.G., Tehan B. (2013). Biophysical fragment screening of the β1-adrenergic receptor: Identification of high affinity arylpiperazine leads using structure-based drug design. J. Med. Chem..

[12] Wachter S.B., Gilbert E.M. (2012). Beta-adrenergic receptors, from their discovery and characterization through their manipulation to beneficial clinical application. Cardiology.

[13] Warne T., Serrano-Vega M.J., Baker J.G., Moukhametzianov R., Edwards P.C., Henderson R., Leslie A.G.W., Tate C.G., Schertler G.F.X. (2008). Structure of a β1-adrenergic G-protein-coupled receptor. Nature.

[14] Katsarou M.-S., Karathanasopoulou A., Andrianopoulou A., Desiniotis V., Tzinis E., Dimitrakis E., Lagiou M., Charmandari E., Aschner M., Tsatsakis A.M. (2018). Beta 1, Beta 2 and Beta 3 Adrenergic Receptor Gene Polymorphisms in a Southeastern European Population. Front. Genet..

[15] Makaritsis K., Triposkiadis F. (2015). Beta adrenergic receptors. Introduction to Translational Cardiovascular Research.

[16] Wang J., Gareri C., Rockman H.A. (2018). G-protein-coupled receptors in heart disease. Circ. Res..

[17] Lymperopoulos A., McCrink K.A., Maning J., Brill A., Desimine V.L., Wertz S.L., Koch W. (2018). Carvedilol Exerts Positive Inotropy in Cardiomyocytes By Uniquely Stimulating Beta-Arrestin2-Dependent Serca2a Activity Via the Beta1-Adrenergic Receptor. J. Am. Coll. Cardiol..

[18] Cang X., Yang L., Yang J., Luo C., Zheng M., Yu K., Yang H., Jiang H. (2014). Cholesterol-β1AR interaction versus cholesterol-β2AR interaction. Proteins Struct. Funct. Bioinform..

[19] Gardner L.A., Tavalin S.J., Goehring A.S., Scott J.D., Bahouth S.W. (2006). AKAP79-mediated targeting of the cyclic AMP-dependent protein kinase to the β1-adrenergic receptor promotes recycling and functional resensitization of the receptor. J. Biol. Chem..

[20] Goth C.K., Tuhkanen H.E., Khan H., Lackman J.J., Wang S., Narimatsu Y., Hansen L.H., Overall C.M., Clausen H., Schjoldager K.T. (2017). Site-specific O-glycosylation by polypeptide N-acetylgalactosaminyltransferase 2 (GalNAc-transferase T2) co-regulates β1-adrenergic receptor N-terminal cleavage. J. Biol. Chem..

[21] Schwalbe T., Huebner H., Gmeiner P. (2019). Development of covalent antagonists for β1- and β2-adrenergic receptors. Bioorganic Med. Chem..

[22] Shan J., Weinstein H., Mehler E.L. (2010). Probing the structural determinants for the function of intracellular loop 2 in structurally cognate G-protein-coupled receptors. Biochemistry.

[23] Vanni S., Neri M., Tavernelli I., Rothlisberger U. (2011). Predicting novel binding modes of agonists to β adrenergic receptors using all-atom molecular dynamics simulations. PLoS Comput. Biol..

[24] Frei J.N., Broadhurst R.W., Bostock M.J., Solt A., Jones A.J.Y., Gabriel F., Tandale A., Shrestha B., Nietlispach D. (2020). Conformational plasticity of ligand-bound and ternary GPCR complexes studied by 19F NMR of the β1-adrenergic receptor. Nat. Commun..

[25] Yohannan S., Faham S., Yang D., Whitelegge J.P., Bowie J.U. (2004). The evolution of transmembrane helix kinks and the structural diversity of G protein-coupled receptors. Proc. Natl. Acad. Sci. USA.

[26] Kim S., Chen J., Cheng T., Gindulyte A., He J., He S., Li Q., Shoemaker B.A., Thiessen P.A., Yu B. (2021). PubChem in 2021: New data content and improved web interfaces. Nucleic Acids Res..

[27] Mysinger M.M., Carchia M., Irwin J.J., Shoichet B.K. (2012). Directory of useful decoys, enhanced (DUD-E): Better ligands and decoys for better benchmarking. J. Med. Chem..

[28] Yap C.W. (2011). PaDEL-descriptor: An open source software to calculate molecular descriptors and fingerprints. J. Comput. Chem..

[29] National Center for Biotechnology Information PubChem Compound Summary for CID 58150421. https://pubchem.ncbi.nlm.nih.gov/compound/4-Nitroaniline#section=NIOSH-Toxicity-Data%0Ahttps://pubchem.ncbi.nlm.nih.gov/compound/58150421.

[30] Salentin S., Schreiber S., Haupt V.J., Adasme M.F., Schroeder M. (2015). PLIP: Fully automated protein-ligand interaction profiler. Nucleic Acids Res..

[31] Ghabbour H.A., El-Bendary E.R., El-Ashmawy M.B., El-Kerdawy M.M. (2014). Synthesis, docking study and β-Adrenoceptor activity of some new oxime ether derivatives. Molecules.

[32] Bai Q., Shao Y., Pan D., Zhang Y., Liu H., Yao X. (2014). Search for β2adrenergic receptor ligands by virtual screening via grid computing and investigation of binding modes by docking and molecular dynamics simulations. PLoS ONE.

[33] Kolb P., Rosenbaum D.M., Irwin J.J., Fung J.J., Kobilka B.K., Shoichet B.K. (2009). Structure-based discovery of β 2 -adrenergic receptor ligands. Proc. Natl. Acad. Sci. USA.

[34] Yang Z., Lu Z.Q., Zhang Y.J., Li Y.B., Wang Z.Y., Zhang Y.L., Zhuang P.W., Bai G. (2016). Looking for agonists of β2adrenergic receptor from Fuzi and Chuanwu by virtual screening and dual-luciferase reporter assay. J. Asian Nat. Prod. Res..

[35] Bojarska J., Remko M., Breza M., Madura I.D., Kaczmarek K., Zabrocki J., Wolf W.M. (2020). A supramolecular approach to structure-based design with a focus on synthons hierarchy in ornithine-derived ligands: Review, synthesis, experimental and in silico studies. Molecules.

[36] Tamura S., Yang G.M., Yasueda N., Matsuura Y., Komoda Y., Murakami N. (2010). Tellimagrandin I, HCV invasion inhibitor from Rosae Rugosae Flos. Bioorganic Med. Chem. Lett..

[37] Tan Y., Ren Y., Gao L., Li L., Cui L., Li B., Li X., Yang J., Wang M., Lv Y. (2018). 28-Day Oral Chronic Toxicity Study of Arctigenin in Rats. Front. Pharmacol..

[38] Baig M.H., Sudhakar D.R., Kalaiarasan P., Subbarao N., Wadhawa G., Lohani M., Khan M.K.A., Khan A.U. (2014). Insight into the effect of inhibitor resistant S130G mutant on physico-chemical properties of SHV type beta-lactamase: A molecular dynamics study. PLoS ONE.

[39] Maia E.H.B., Assis L.C., de Oliveira T.A., da Silva A.M., Taranto A.G. (2020). Structure-Based Virtual Screening: From Classical to Artificial Intelligence. Front. Chem..

[40] Maia E.H.B., Medaglia L.R., Da Silva A.M., Taranto A.G. (2020). Molecular Architect: A User-Friendly Workflow for Virtual Screening. ACS Omega.

[41] Neves B.J., Braga R.C., Melo-Filho C.C., Moreira-Filho J.T., Muratov E.N., Andrade C.H. (2018). QSAR-based virtual screening: Advances and applications in drug discovery. Front. Pharmacol..

[42] Liu C., Yin J., Yao J., Xu Z., Tao Y., Zhang H. (2020). Pharmacophore-Based Virtual Screening toward the Discovery of Novel Anti-echinococcal Compounds. Front. Cell. Infect. Microbiol..

[43] Durán Á., Zamora I., Pastor M. (2009). Suitability of GRIND-based principal properties for the description of molecular similarity and ligand-based virtual screening. J. Chem. Inf. Model..

[44] Stahura F., Bajorath J. (2005). New Methodologies for Ligand-Based Virtual Screening. Curr. Pharm. Des..

[45] Andricopulo A., Guido R., Oliva G. (2008). Virtual Screening and Its Integration with Modern Drug Design Technologies. Curr. Med. Chem..

[46] Wang Z., Sun H., Shen C., Hu X., Gao J., Li D., Cao D., Hou T. (2020). Combined strategies in structure-based virtual screening. Phys. Chem. Chem. Phys..

[47] Maffucci I., Contini A. (2013). Explicit ligand hydration shells improve the correlation between MM-PB/GBSA binding energies and experimental activities. J. Chem. Theory Comput..

[48] Ferreira L.G., Dos Santos R.N., Oliva G., Andricopulo A.D. (2015). Molecular docking and structure-based drug design strategies. Molecules.

[49] Waszkowycz B., Clark D.E., Gancia E. (2011). Outstanding challenges in protein-ligand docking and structure-based virtual screening. Wiley Interdiscip. Rev. Comput. Mol. Sci..

[50] Cavasotto C.N., W Orry A.J. (2007). Ligand Docking and Structure-based Virtual Screening in Drug Discovery. Curr. Top. Med. Chem..

[51] Kitchen D.B., Decornez H., Furr J.R., Bajorath J. (2004). Docking and scoring in virtual screening for drug discovery: Methods and applications. Nat. Rev. Drug Discov..

[52] Xu L., Zhang Y., Zheng L., Qiao C., Li Y., Li D., Zhen X., Hou T. (2014). Discovery of novel inhibitors targeting the macrophage migration inhibitory factor via structure-based virtual screening and bioassays. J. Med. Chem..

[53] Wu J.S., Lin S.Y., Liao F.Y., Hsiao W.C., Lee L.C., Peng Y.H., Hsieh C.L., Wu M.H., Song J.S., Yueh A. (2015). Identification of Substituted Naphthotriazolediones as Novel Tryptophan 2,3-Dioxygenase (TDO) Inhibitors through Structure-Based Virtual Screening. J. Med. Chem..

[54] Smith E.W., Nevins A.M., Qiao Z., Liu Y., Getschman A.E., Vankayala S.L., Kemp M.T., Peterson F.C., Li R., Volkman B.F. (2016). Structure-Based Identification of Novel Ligands Targeting Multiple Sites within a Chemokine-G-Protein-Coupled-Receptor Interface. J. Med. Chem..

[55] Gupta S., Parihar D., Shah M., Yadav S., Managori H., Bhowmick S., Patil P.C., Alissa S.A., Wabaidur S.M., Islam M.A. (2020). Computational screening of promising beta-secretase 1 inhibitors through multi-step molecular docking and molecular dynamics simulations—Pharmacoinformatics approach. J. Mol. Struct..

[56] Choudhary S., Malik Y.S., Tomar S. (2020). Identification of SARS-CoV-2 Cell Entry Inhibitors by Drug Repurposing Using in silico Structure-Based Virtual Screening Approach. Front. Immunol..

[57] Sinha S.K., Prasad S.K., Islam M.A., Gurav S.S., Patil R.B., AlFaris N.A., Aldayel T.S., AlKehayez N.M., Wabaidur S.M., Shakya A. (2020). Identification of bioactive compounds from Glycyrrhiza glabra as possible inhibitor of SARS-CoV-2 spike glycoprotein and non-structural protein-15: A pharmacoinformatics study. J. Biomol. Struct. Dyn..

[58] Shetve V.V., Bhowmick S., Alissa S.A., Alothman Z.A., Wabaidu S.M., Asmary F.A., Alhajri H.M., Islam M.A. (2021). Identification of selective Lyn inhibitors from the chemical databases through integrated molecular modelling approaches. SAR QSAR Environ. Res..

[59] Bolton E.E., Wang Y., Thiessen P.A., Bryant S.H. (2008). Chapter 12 PubChem: Integrated Platform of Small Molecules and Biological Activities. Annual Reports in Computational Chemistry.

[60] Wang Y., Xiao J., Suzek T.O., Zhang J., Wang J., Bryant S.H. (2009). PubChem: A public information system for analyzing bioactivities of small molecules. Nucleic Acids Res..

[61] Agarwala R., Barrett T., Beck J., Benson D.A., Bollin C., Bolton E., Bourexis D., Brister J.R., Bryant S.H., Canese K. (2018). Database resources of the National Center for Biotechnology Information. Nucleic Acids Res..

[62] Kim S., Thiessen P.A., Bolton E.E., Chen J., Fu G., Gindulyte A., Han L., He J., He S., Shoemaker B.A. (2016). PubChem substance and compound databases. Nucleic Acids Res..

[63] Lipinski C.A. (2004). Lead- and drug-like compounds: The rule-of-five revolution. Drug Discov. Today Technol..

[64] Ghose A.K., Viswanadhan V.N., Wendoloski J.J. (1999). A knowledge-based approach in designing combinatorial or medicinal chemistry libraries for drug discovery. 1. A qualitative and quantitative characterization of known drug databases. J. Comb. Chem..

[65] Veber D.F., Johnson S.R., Cheng H.Y., Smith B.R., Ward K.W., Kopple K.D. (2002). Molecular properties that influence the oral bioavailability of drug candidates. J. Med. Chem..

[66] Congreve M., Carr R., Murray C., Jhoti H. (2003). A “Rule of Three” for fragment-based lead discovery?. Drug Discov. Today.

[67] Schneider G. (2002). Prediction of Drug-Like Properties. Adaptive Systems in Drug Design.

[68] Landrum G. RDKit: Open-Source Cheminformatics Software. http://www.Rdkit.Org/2021.

[69] O′Boyle N.M., Banck M., James C.A., Morley C., Vandermeersch T., Hutchison G.R. (2011). Open Babel: An Open chemical toolbox. J. Cheminform..

[70] Trott O., Olson A.J. (2009). AutoDock Vina: Improving the speed and accuracy of docking with a new scoring function, efficient optimization, and multithreading. J. Comput. Chem..

[71] Berman H.M., Westbrook J., Feng Z., Gilliland G., Bhat T.N., Weissig H., Shindyalov I.N., Bourne P.E. (2000). The Protein Data Bank. Nucleic Acids Res..

[72] Zardecki C., Dutta S., Goodsell D.S., Voigt M., Burley S.K. (2016). RCSB Protein Data Bank: A Resource for Chemical, Biochemical, and Structural Explorations of Large and Small Biomolecules. J. Chem. Educ..

[73] Cherezov V., Rosenbaum D.M., Hanson M., Rasmussen S.G., Choi H.-J., Kuhn P., Weis W., Kobika B., Stevens R.C. (2007). High-Resolution Crystal Structure of an Engineered Human β2- Adrenergic G Protein–Coupled Receptor. Science.

[74] Stanzione F., Giangreco I., Cole J.C. (2021). Use of molecular docking computational tools in drug discovery. Progress in Medicinal Chemistry.

[75] Nguyen N.T., Nguyen T.H., Pham T.N.H., Huy N.T., Bay M., Van Pham M.Q., Nam P.C., Vu V.V., Ngo S.T. (2020). Autodock Vina Adopts More Accurate Binding Poses but Autodock4 Forms Better Binding Affinity. J. Chem. Inf. Model..

[76] Steffen C., Thomas K., Huniar U., Hellweg A., Rubner O., Schroer A. (2010). Software news and updates TmoleX-a graphical user interface for TURBOMOLE. J. Comput. Chem..

[77] Gaillard T. (2018). Evaluation of AutoDock and AutoDock Vina on the CASF-2013 Benchmark. J. Chem. Inf. Model..

[78] Sutherland J.J., Nandigam R.K., Erickson J.A., Vieth M. (2007). Lessons in molecular recognition. 2. Assessing and improving cross-docking accuracy. J. Chem. Inf. Model..

[79] Hevener K.E., Zhao W., Ball D.M., Babaoglu K., Qi J., White S.W., Lee R.E. (2009). Validation of molecular docking programs for virtual screening against dihydropteroate synthase. J. Chem. Inf. Model..

[80] Carlberg B., Samuelsson O., Lindholm P.L.H. (2004). Atenolol in hypertension: Is it a wise choice?. Lancet.

[81] Wishart D.S., Feunang Y.D., Guo A.C., Lo E.J., Marcu A., Grant J.R., Sajed T., Johnson D., Li C., Sayeeda Z. (2018). DrugBank 5.0: A major update to the DrugBank database for 2018. Nucleic Acids Res..

[82] Wishart D.S., Knox C., Guo A.C., Shrivastava S., Hassanali M., Stothard P., Chang Z., Woolsey J. (2006). DrugBank: A comprehensive resource for in silico drug discovery and exploration. Nucleic Acids Res..

[83] Gaulton A., Hersey A., Nowotka M.L., Patricia Bento A., Chambers J., Mendez D., Mutowo P., Atkinson F., Bellis L.J., Cibrian-Uhalte E. (2017). The ChEMBL database in 2017. Nucleic Acids Res..

[84] Willighagen E.L., Waagmeester A., Spjuth O., Ansell P., Williams A.J., Tkachenko V., Hastings J., Chen B., Wild D.J. (2013). The ChEMBL database as linked open data. J. Cheminform..

[85] Cai C., Wang S., Xu Y., Zhang W., Tang K., Ouyang Q., Lai L., Pei J. (2020). Transfer Learning for Drug Discovery. J. Med. Chem..

[86] Wu Z., Ramsundar B., Feinberg E.N., Gomes J., Geniesse C., Pappu A.S., Leswing K., Pande V. (2018). MoleculeNet: A benchmark for molecular machine learning. Chem. Sci..

[87] Ma J., Sheridan R.P., Liaw A., Dahl G.E., Svetnik V. (2015). Deep neural nets as a method for quantitative structure-activity relationships. J. Chem. Inf. Model..

[88] Yang K., Swanson K., Jin W., Coley C., Eiden P., Gao H., Guzman-Perez A., Hopper T., Kelley B., Mathea M. (2019). Analyzing Learned Molecular Representations for Property Prediction. J. Chem. Inf. Model..

[89] Ragoza M., Hochuli J., Idrobo E., Sunseri J., Koes D.R. (2017). Protein-Ligand Scoring with Convolutional Neural Networks. J. Chem. Inf. Model..

[90] Wang C., Zhang Y. (2017). Improving scoring-docking-screening powers of protein–ligand scoring functions using random forest. J. Comput. Chem..

[91] Segler M.H.S., Preuss M., Waller M.P. (2018). Planning chemical syntheses with deep neural networks and symbolic AI. Nature.

[92] Coley C.W., Thomas D.A., Lummiss J.A.M., Jaworski J.N., Breen C.P., Schultz V., Hart T., Fishman J.S., Rogers L., Gao H. (2019). A robotic platform for flow synthesis of organic compounds informed by AI planning. Science.

[93] Xu Y., Lin K., Wang S., Wang L., Cai C., Song C., Lai L., Pei J. (2019). Deep learning for molecular generation. Future Med. Chem..

[94] Elton D.C., Boukouvalas Z., Fuge M.D., Chung P.W. (2019). Deep learning for molecular design—A review of the state of the art. Mol. Syst. Des. Eng..

[95] Li H., Sze K.H., Lu G., Ballester P.J. (2020). Machine-learning scoring functions for structure-based drug lead optimization. Wiley Interdiscip. Rev. Comput. Mol. Sci..

[96] Quinlan J.R. (1987). Simplifying decision trees. Int. J. Man. Mach. Stud..

[97] Ho T.K. Random decision forests. Proceedings of the International Conference on Document Analysis and Recognition, ICDAR 1995.

[98] Walker S.H., Duncan D.B. (1967). Estimation of the probability of an event as a function of several independent variables. Biometrika.

[99] Friedman J.H. (2001). Greedy function approximation: A gradient boosting machine. Ann. Stat..

[100] Altman N.S. (1992). An introduction to kernel and nearest-neighbor nonparametric regression. Am. Stat..

[101] Cortes C., Vapnik V. (1995). Support-Vector Networks. Mach. Learn..

[102] Pedregosa F., Varoquaux G., Gramfort A., Michel V., Thirion B., Grisel O., Blondel M., Prettenhofer P., Weiss R., Dubourg V. (2011). Scikit-learn: Machine learning in Python. J. Mach. Learn. Res..

[103] Daina A., Michielin O., Zoete V. (2017). SwissADME: A free web tool to evaluate pharmacokinetics, drug-likeness and medicinal chemistry friendliness of small molecules. Sci. Rep..

[104] Pires D.E.V., Blundell T.L., Ascher D.B. (2015). pkCSM: Predicting small-molecule pharmacokinetic and toxicity properties using graph-based signatures. J. Med. Chem..

[105] Edgar S.J., Holliday J.D., Willett P. (2000). Effectiveness of retrieval in similarity searches of chemical databases: A review of performance measures. J. Mol. Graph. Model..

[106] Bender A., Glen R.C. (2004). Molecular similarity: A key technique in molecular informatics. Org. Biomol. Chem..

[107] Yu X., Geer L.Y., Han L., Bryant S.H. (2015). Target enhanced 2D similarity search by using explicit biological activity annotations and profiles. J. Cheminform..

[108] Rogers D.J., Tanimoto T.T. (1960). A computer program for classifying plants. Science.

[109] Bajusz D., Rácz A., Héberger K. (2015). Why is Tanimoto index an appropriate choice for fingerprint-based similarity calculations?. J. Cheminform..

[110] Lindahl A., Hess S.V.D., van der Spoel D. (2021). GROMACS 2021.3 Source code.

[111] Jo S., Kim T., Iyer V.G., Im W. (2008). CHARMM-GUI: A web-based graphical user interface for CHARMM. J. Comput. Chem..

[112] Mark P., Nilsson L. (2001). Structure and dynamics of the TIP3P, SPC, and SPC/E water models at 298 K. J. Phys. Chem. A.

[113] Joung I.S., Cheatham T.E. (2008). Determination of alkali and halide monovalent ion parameters for use in explicitly solvated biomolecular simulations. J. Phys. Chem. B.

[114] He X., Man V.H., Yang W., Lee T.S., Wang J. (2020). A fast and high-quality charge model for the next generation general AMBER force field. J. Chem. Phys..

