# Supplementary material for: Screening of β1- and β2-Adrenergic Receptor Modulators through Advanced Pharmacoinformatics and Machine Learning Approaches
Source: Int J Mol Sci. 2021 Oct 17;22(20):11191. doi: 10.3390/ijms222011191 (PMC8538848; doi:10.3390/ijms222011191)
Supplement: Supplementary file 1 [file ijms-22-11191-s001.zip › ijms-1404869-supplementary.pdf]

Supplementary data

Screening of β1- and β2-adrenergic receptor modulators through advanced pharmacoinformatics and machine learning approaches

Table S1. Predicted toxicity properties of final proposed molecules for β1- and β2-AR

Toxicity properties	β1-AR				β2-AR			
	**M1**	**M2**	**M3**	**M4**	**M5**	**M6**	**M7**	**M8**
AMES toxicity	No	No	No	No	No	Yes	Yes	Yes
Max. tolerated dose (human)	0.05	-0.18	-0.12	0.09	-0.25	-0.20	-0.01	-0.88
hERG I/hERG II inhibitor	No / No	No / No	No / No	No / No	No / No	No / Yes	No / No	No / No
Oral Rat Acute Toxicity (LD_{50})	2.67	2.18	2.73	2.26	3.14	2.64	2.62	3.29
Oral Rat Chronic Toxicity (LOAEL)	1.19	1.65	0.54	0.85	0.95	0.82	1.10	1.28
Hepatotoxicity	No	No	No	No	No	No	No	Yes
Skin Sensitisation	No	No	No	No	No	No	No	No
T.Pyriformis toxicity	0.57	0.44	0.31	0.46	0.43	0.84	0.72	0.28
Minnow toxicity	1.04	0.54	1.44	1.78	1.40	0.51	0.52	0.44

M1: PubChem_21122992; M2: PubChem_26183498; M3: PubChem_8766520; M4: PubChem_153007611; M5: PubChem_498002; M6: PubChem_3880315; M7: PubChem_12308663 and M8: PubChem_151341014

Table S2. Statistical parameters of beta-1 and beta-2AR obtained from MD simulation trajectories

		β1-AR					β2-AR				
		M1	M2	M3	M4	M5	M1	M6	M7	M8	M9
RMSD (nm)	Min.	0.000	0.000	0.000	0.000	0.000	0.000	0.000	0.000	0.000	0.000
	Max.	0.216	0.281	0.250	0.308	0.256	0.977	0.627	0.894	0.618	0.663
	Avg.	0.154	0.196	0.187	0.223	0.177	0.532	0.372	0.533	0.473	0.450
RMSD-ligand (nm)	Min.	0.000	0.000	0.000	0.000	0.000	0.000	0.000	0.000	0.000	0.000
	Max.	0.216	0.075	0.039	0.062	0.058	0.239	0.038	0.142	0.109	0.141
	Avg.	0.154	0.029	0.022	0.029	0.032	0.149	0.016	0.077	0.084	0.034
RMSF (nm)	Min.	0.049	0.050	0.050	0.049	0.053	0.666	0.449	0.749	0.454	0.551
	Max.	0.309	0.386	0.303	0.461	0.361	0.084	0.061	0.084	0.079	0.081
	Avg.	0.108	0.116	0.118	0.124	0.123	0.271	0.188	0.324	0.188	0.222
RoG (nm)	Min.	2.050	2.070	2.064	2.059	2.064	2.760	2.843	2.845	2.800	2.919
	Max.	2.075	2.123	2.094	2.110	2.089	3.009	2.992	3.105	3.037	3.086
	Avg.	2.027	2.033	2.037	2.018	2.043	2.877	2.914	2.961	2.923	3.004

Min.: Minimum; Max.: Maximum; Avg. Average; M1: Atenolol; M2: PubChem_21122992; M3: PubChem_26183498; M4: PubChem_8766520; M5: PubChem_153007611; M6: PubChem_498002; M7: PubChem_3880315; M8: PubChem_12308663 and M9: PubChem_151341014

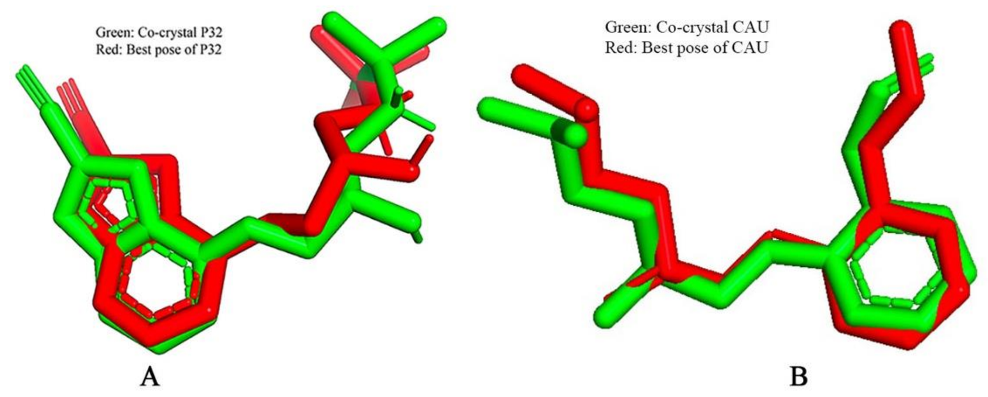

Figure S1. Superimposition of co-crystal and best docked pose of P32 and CAU .

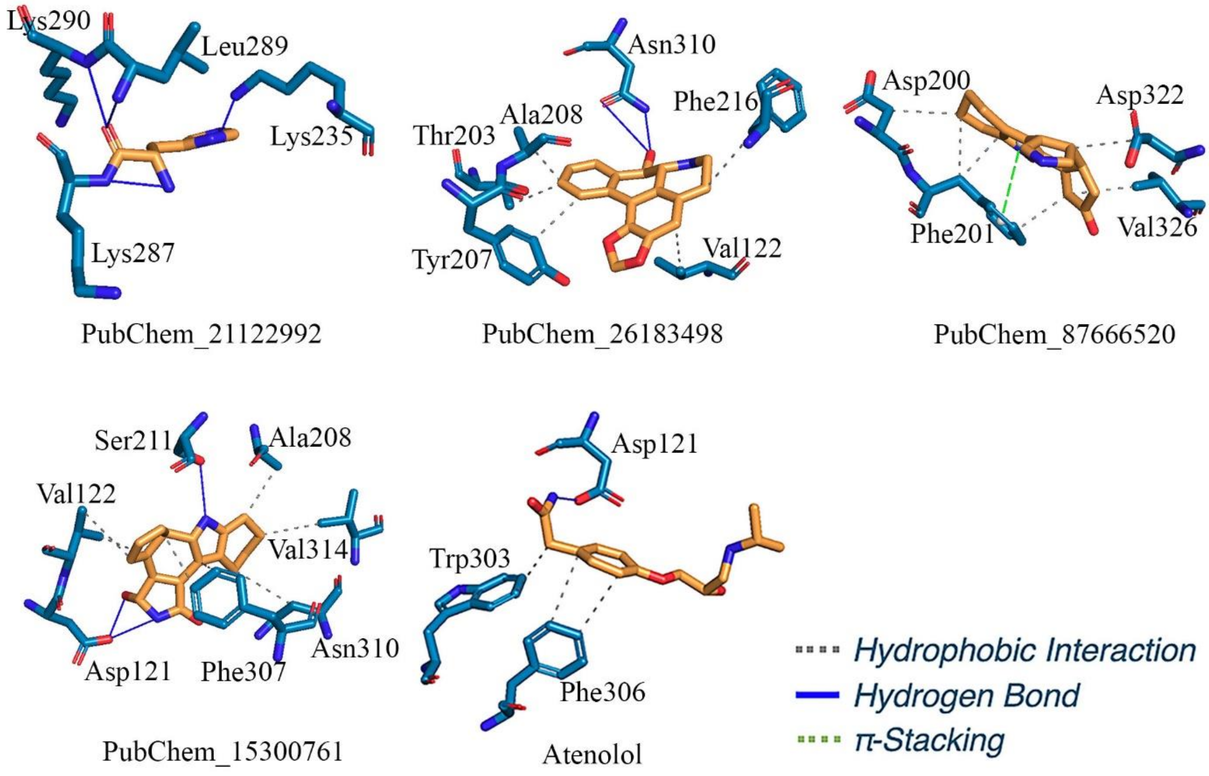

Figure S2. Post-MD simulation binding interaction profile of β1-AR molecules.

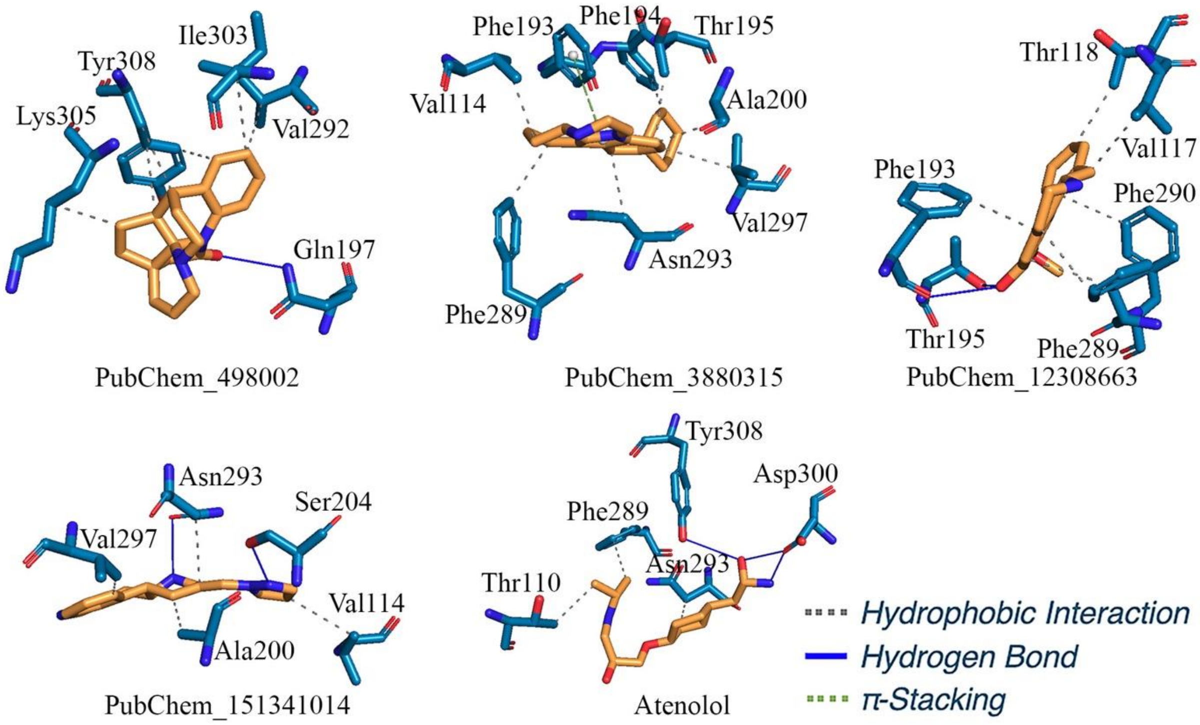

Figure S3. Post-MD simulation binding interaction profile of β2-AR molecules.

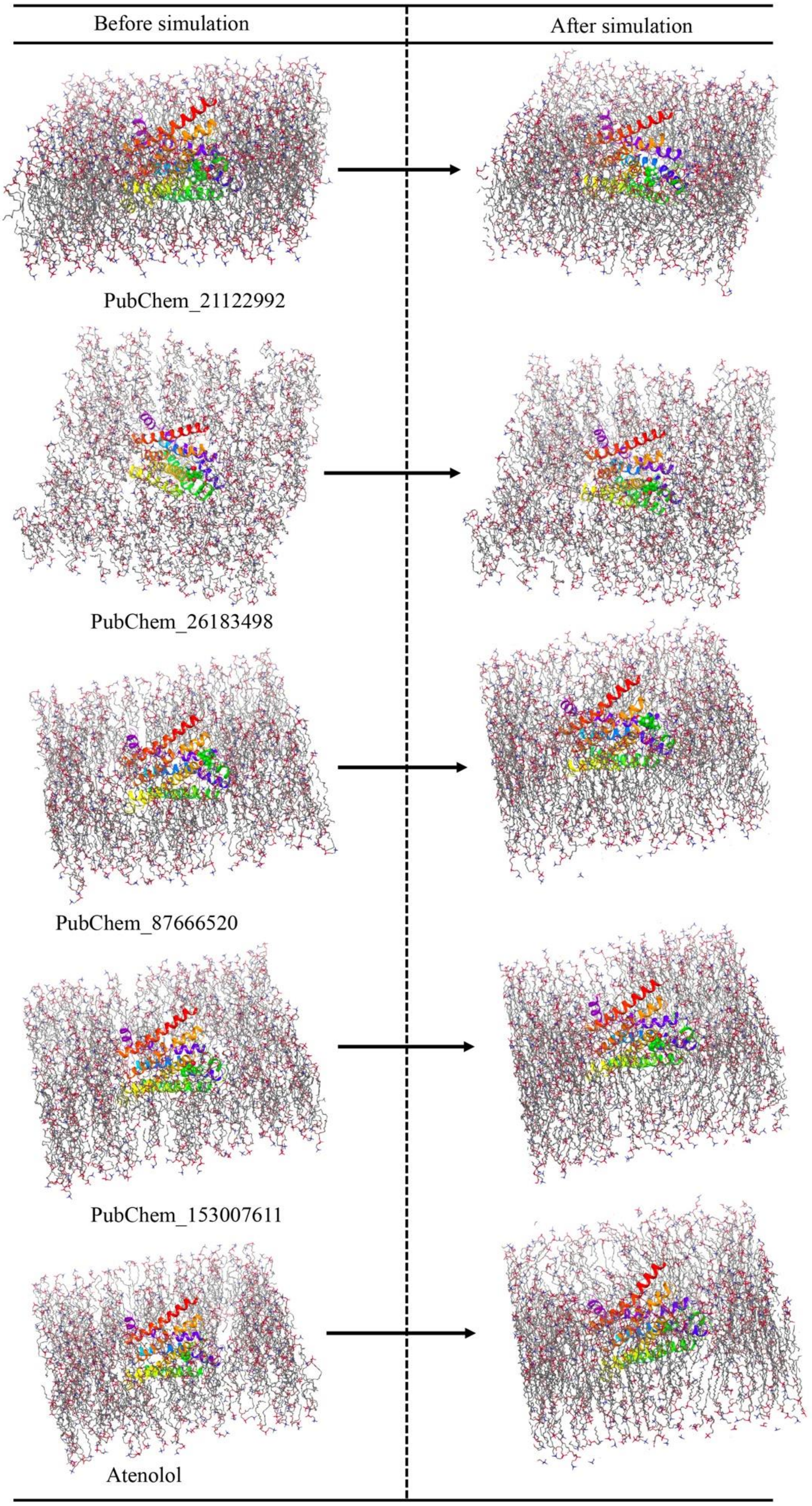

Figure S4. Protein-ligand complex of β1-AR inside the lipid bilayer before and after simulation

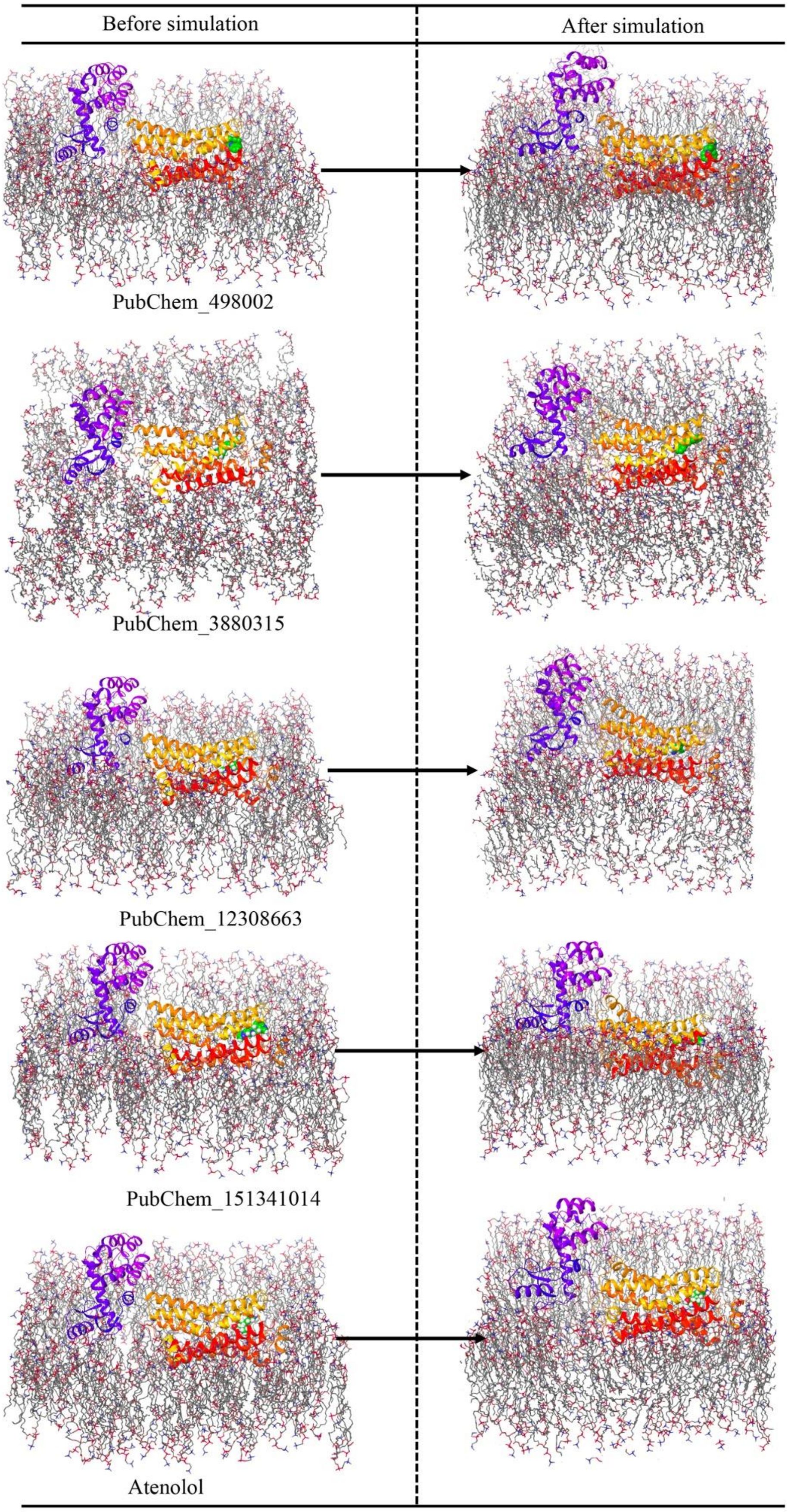

Figure S5. Protein-ligand complex of β2-AR inside the lipid bilayer before and after simulation
